# A fungal effector and a rice NLR protein have antagonistic effects on a Bowman–Birk trypsin inhibitor

**DOI:** 10.1111/pbi.13400

**Published:** 2020-07-04

**Authors:** Chongyang Zhang, Hong Fang, Xuetao Shi, Feng He, Ruyi Wang, Jiangbo Fan, Pengfei Bai, Jiyang Wang, Chan‐Ho Park, Maria Bellizzi, Xueping Zhou, Guo‐Liang Wang, Yuese Ning

**Affiliations:** ^1^ State Key Laboratory for Biology of Plant Diseases and Insect Pests Institute of Plant Protection Chinese Academy of Agricultural Sciences Beijing 100193 China; ^2^ Department of Plant Pathology Ohio State University Columbus OH 43210 USA

**Keywords:** *Magnaporthe oryzae* effector, rice, Bowman–Birk trypsin inhibitor, NLR protein, plant immunity

## Abstract

Bowman–Birk trypsin inhibitors (BBIs) play important roles in animal and plant immunity, but how these protease inhibitors are involved in the immune system remains unclear. Here, we show that the rice (*Oryza sativa*) BBI protein APIP4 is a common target of a fungal effector and an NLR receptor for innate immunity. APIP4 exhibited trypsin inhibitor activity *in vitro* and *in vivo*. Knockout of *APIP4* in rice enhanced susceptibility, and overexpression of *APIP4* increased resistance to the fungal pathogen *Magnaporthe oryzae*. The *M. oryzae* effector AvrPiz‐t interacted with APIP4 and suppressed APIP4 trypsin inhibitor activity. By contrast, the rice NLR protein Piz‐t interacted with APIP4, enhancing APIP4 transcript and protein levels, and protease inhibitor activity. Our findings reveal a novel host defence mechanism in which a host protease inhibitor targeted by a fungal pathogen is protected by an NLR receptor.

## Introduction

Phytopathogens have evolved multiple strategies to attack plants, including the secretion of effector proteins into host cells. During their coevolution with pathogens, plants have developed receptors and other proteins that recognize pathogen factors to trigger immune responses (Jones and Dangl, 2006; Jones *et al*., 2016). Pathogens and plants both employ proteases and protease inhibitors to modulate invasion and defence, respectively (Hou *et al*., 2018). Some pathogen effectors inhibit host proteases, thus subverting plant immunity (Hou *et al*., 2018; Misas‐Villamil and Hoorn, 2008). For instance, the potato (*Solanum tuberosum*) late blight pathogen *Phytophthora infestans* secretes two divergent Kazal‐like protease inhibitors, EPI1 and EPI10, which target the tomato (*Lycopersicon esculentum*) subtilase P69B (Tian *et al*., 2005; Tian *et al*., 2004). Similarly, the cystatin‐like protease inhibitors EPIC1 and EPIC2B from *P. infestans* target the tomato defence proteases C14 and Rcr3^pim^ (Kaschani *et al*., 2010; Song *et al*., 2009). In addition, the *Ustilago maydis* effector protein Pit2 inhibits a set of maize (*Zea mays*) cysteine proteases to undermine host resistance (Mueller *et al*., 2013). These studies indicate that the suppression of host proteases by pathogen inhibitors is a common strategy to subvert host immunity. By contrast, only a few studies have demonstrated that pathogen proteases target host protease inhibitors during infection. For example, the *P. sojae* apoplastic effector PsXEG1 targets a soya bean (*Glycine max*) apoplastic glucanase inhibitor protein, GmGIP1, to impair infection (Ma *et al*., 2016). To protect PsXEG1 from inhibition by GmGIP1 in the apoplast, *P. sojae* secretes PsXLP1, a paralog of PsXEG1 (Ma *et al*., 2016). However, it is unknown whether the cytoplasmic effectors from pathogens target host protease inhibitors to subvert immunity.

Although proteases and protease inhibitors are involved in pathogen‐associated molecular pattern‐triggered immunity or basal resistance against pathogens (Chen *et al*., 2014; Hoorn, 2008; Quilis *et al*., 2014), only a few studies have reported their functions in resistance (R) protein‐mediated immunity. A well‐studied example comes from research on the interaction between the tomato R protein Cf‐2 and its cognate effector Avr2 from the fungal pathogen *Cladosporium fulvum* (Rooney. *et al*., 2005). Avr2 physically interacts with the protease Rcr3 and inhibits its activity. Absence of Rcr3 activity in *rcr3* mutants does not trigger the resistance response mediated by Cf‐2 (Rooney. *et al*., 2005), suggesting that the R protein Cf‐2 guards the virulence target Rcr3 of the effector Avr2. Another example is the *Arabidopsis* cysteine protease RD19, which re‐localizes to the nucleus after interacting with the *Ralstonia solanacearum* effector PopP2. PopP2 and RD19 form a complex in the nucleus to activate the RRS1‐mediated resistance response (Bernoux *et al*., 2008). However, it has not yet been reported that a protease inhibitor is the target of an avirulence effector and is modulated by the corresponding R protein in plants.

The Bowman–Birk trypsin inhibitors (BBIs) are widely known in soya bean and belong to the serpin superfamily of protease inhibitors, which possess trypsin and chymotrypsin activity (Birk, 1985). A growing body of evidence suggests that BBIs act as anticancer and chemopreventive agents by suppressing cancer cell proliferation (Fereidunian *et al*., 2014). Studies in animal models have proven that dietary BBIs from several legume species, including soya bean, pea (*Vicia faba*), lentil (*Lens culinaris*) and chickpea (*Cicer arietinum*), can prevent or suppress carcinogenic and inflammatory processes within the gastrointestinal tract (Clemente and Arques Mdel, 2014). Moreover, BBIs from plants such as wheat (*Triticum aestivum*) and maize can strongly inhibit the growth of plant pathogenic fungi (Chen *et al*., 1999; Chilosi *et al*., 2000). These results suggest that the BBIs play an important role in animal and plant immunity.

Rice blast, caused by the fungal pathogen *Magnaporthe oryzae,* is a devastating disease of rice, a crop that feeds half the world’s population (Talbot, 2003; Wang and Valent, 2017). *Piz‐t* encodes a nucleotide‐binding and leucine‐rich repeat (NLR) *R* protein that confers broad‐spectrum disease resistance and is widely used in rice breeding (Wu *et al*., 2017; Zhou *et al*., 2006). The effector AvrPiz‐t from *M. oryzae* is the corresponding avirulence effector of Piz‐t (Li *et al*., 2009), but these two proteins do not interact directly (Park *et al*., 2012). In this study, we report that the *M. oryzae* effector AvrPiz‐t targets and inhibits the activity of the rice BBI APIP4 (AvrPiz‐t Interacting Protein 4) to facilitate infection. Interestingly, the rice NLR protein Piz‐t interacts with APIP4 and enhances its accumulation and trypsin inhibitor activity. Our study thus revealed a novel mechanism in which a fungal pathogen effector suppresses a host protease inhibitor to attack the plant immune system, and in which an NLR protein weakens this attack by acting on the same protease inhibitor.

## Results

### AvrPiz‐t interacts with APIP4 *in vitro* and *in vivo*

**Figure 1:**
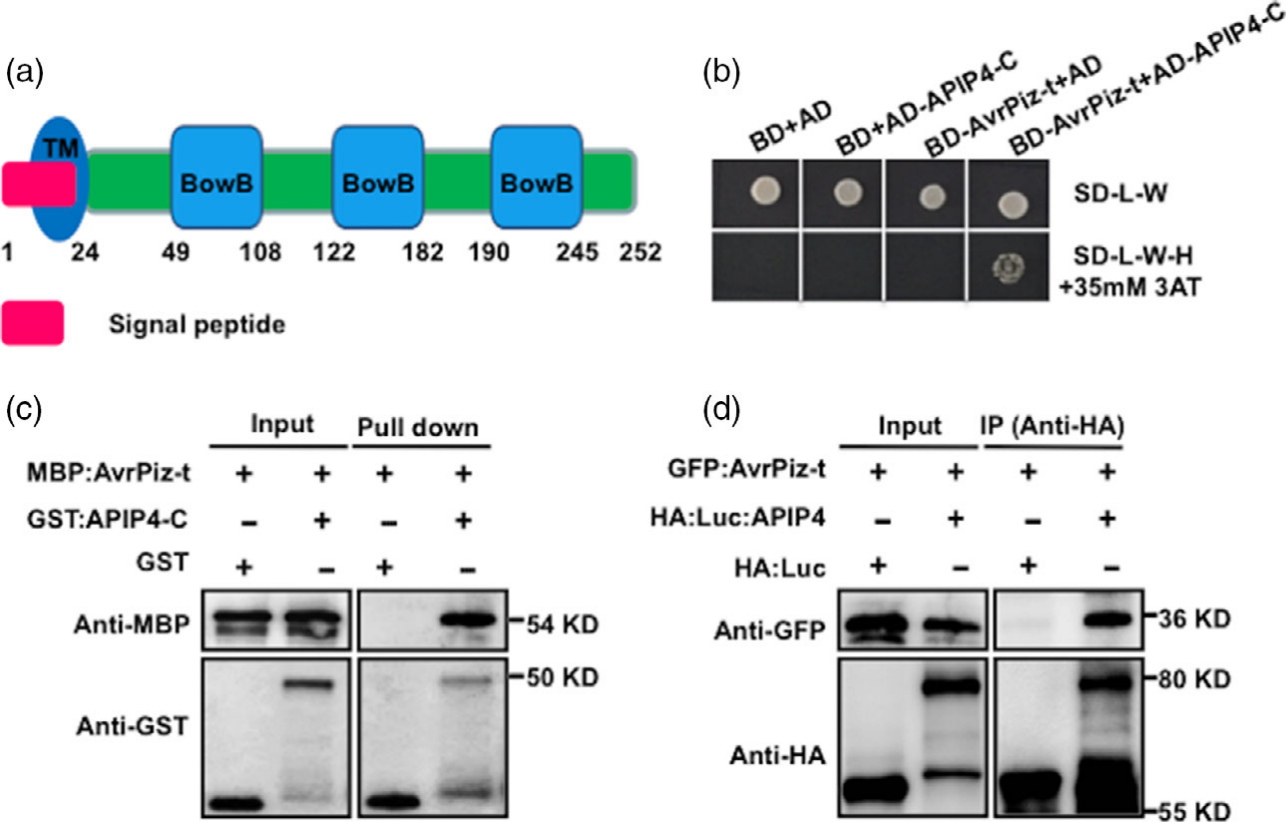
AvrPiz‐t targets APIP4 *in vitro* and *in vivo*. (a) The protein structure of APIP4. The position of the signal peptide, transmembrane domain and three BowB domains is indicated. (b) Interaction of AvrPiz‐t with APIP4‐C in yeast two‐hybrid assay. The growth of yeast colonies on the plate (SD‐L‐W‐H) lacking leucine (L), tryptophan(W) and histidine (H) with 35 mm 3‐aminotriazole (3 AT) indicates a positive interaction. 3 AT is a competitive inhibitor of the *HIS3* gene product (histidine synthase), which is the reporter gene for the interaction in the yeast two‐hybrid assay. (c) Detection of the interaction of AvrPiz‐t and APIP4‐C *in vitro* with a GST pull‐down assay. MBP‐tagged AvrPiz‐t (MBP‐AvrPiz‐t) and GST‐tagged APIP4‐C recombinant proteins were expressed, and the protein–protein interaction was tested by a GST pull‐down assay. (d) Co‐IP assay of AvrPiz‐t and APIP4 in rice protoplasts. The *AvrPiz‐t‐GFP* and *HA‐Luc‐APIP4* plasmids were used for co‐transfection of rice protoplasts. Protein isolated from rice protoplasts was immunoprecipitated with the anti‐HA antibody. Immunoblot analysis was performed using the anti‐HA and anti‐GFP antibodies.

*M. oryzae* AvrPiz‐t is the avirulence effector of Piz‐t in rice (Li *et al*., 2009) and is secreted into the cytoplasm of rice cells at the early stages of infection (Park *et al*., 2012). APIP4 was identified as one of the AvrPiz‐t interacting proteins in a yeast two‐hybrid Y2H screen in our previous study (Park *et al*., 2012). APIP4 contains a signal peptide, transmembrane domain (TM domain) and three BowB domains (Figure 1a). To confirm the interaction between AvrPiz‐t and APIP4, a yeast two‐hybrid assay was conducted using the AvrPiz‐t (without the signal peptide) and APIP4‐C (containing the full intracellular domain) fragments. Colony growth on selective media containing 35 mM 3‐amino‐1,2,4‐triazole (3AT) validated the interaction between AvrPiz‐t and APIP4‐C (Figure 1b). Two unrelated effectors, AvrPi9 and AvrPii (Wu *et al*., 2015; Yoshida *et al*., 2009), did not interact with APIP4‐C (Figure S1a), demonstrating that AvrPiz‐t specifically interacts with APIP4.

Next, we performed a GST pull‐down assay with purified maltose‐binding protein (MBP)‐fused AvrPiz‐t (MBP:AvrPiz‐t) and GST‐fused APIP4‐C (GST:APIP4‐C), as well as the empty control GST. Immunoblot analysis showed that the GST‐fused APIP4‐C proteins bound to MBP‐AvrPiz‐t, whereas GST alone did not (Figure 1c). Finally, we confirmed the interaction between AvrPiz‐t and APIP4 in rice protoplasts using a co‐immunoprecipitation (Co‐IP) assay. Plasmids of GFP:AvrPiz‐t and HA:Luc:APIP4 were co‐transfected into Nipponbare (NPB) rice protoplasts. Immunoblot analysis specifically detected AvrPiz‐t in the APIP4 immunocomplex proteins (Figure 1d, upper panel in the last lane). Overall, these results suggest that AvrPiz‐t specifically interacts with APIP4 *in vitro* and *in vivo*.

AvrPiz‐t was found to be secreted into rice cells during rice infection (Park *et al*., 2016). To determine whether APIP4 is also localized in the cytoplasm, we transfected *APIP4‐GFP* and *mCherry* empty plasmids into rice protoplasts. Fluorescence signals of APIP4‐GFP were clearly observed mainly in the cytoplasm (Figure S2a), indicating that APIP4 is a cytoplasmic protein. The protein level of APIP4 in rice protoplasts was confirmed by immunoblot analysis (Figure S2b). Furthermore, we found that APIP4 had overlapped signals in the cytoplasm with AvrPiz‐t (Figure S2c).

### AvrPiz‐t interferes with APIP4 trypsin inhibitor activity *in vitro* and *in vivo*

**Figure 2:**
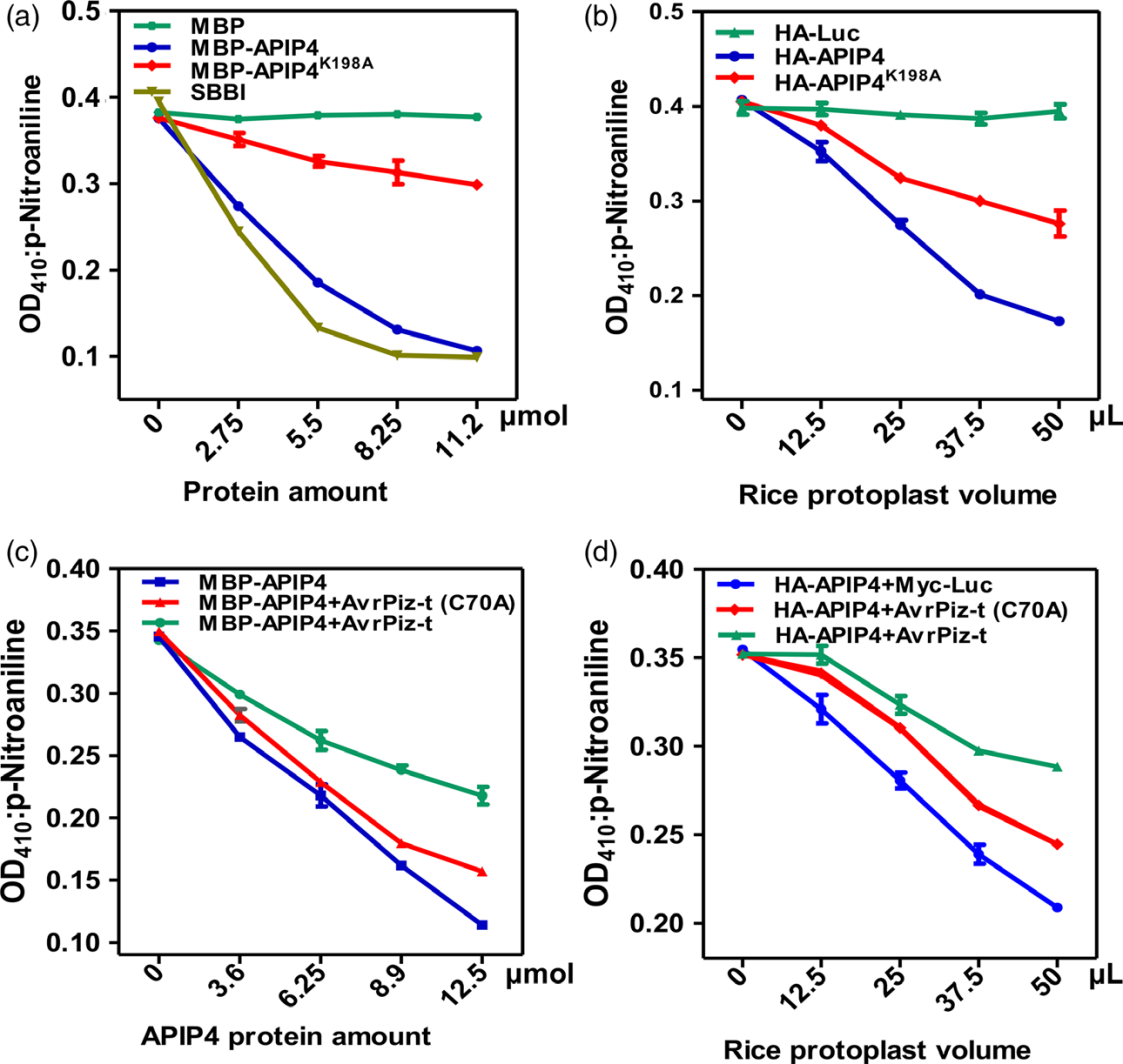
AvrPiz‐t interferes with APIP4 trypsin inhibitor activity *in vitro* and *in vivo*. (a) Trypsin inhibitor activity of APIP4 *in vitro*. The x‐axis indicates the protein amount. The y‐axis indicates the absorbance of p‐nitroaniline, the product of the trypsin in the reaction, at 410 nm. MBP was used as the negative control, and SBBI from soya bean was used as the positive control. (b) Trypsin inhibitor activity assay of APIP4 *in vivo*. HA‐Luc was used as the negative control. (c) Inhibition of APIP4 trypsin inhibitor activity by AvrPiz‐t *in vitro*. A trypsin inhibitor assay was performed by incubating increasing amounts of the APIP4 proteins and trypsin at 37°C for 20 min in the presence of about 12.5 µmol AvrPiz‐t and 12.5 µmol AvrPiz‐t (C70A). The x‐axis shows the protein amounts. (d) Suppression of APIP4 trypsin inhibitor activity by AvrPiz‐t *in vivo*. About 10 µg of plasmids *HA‐APIP4* and *Myc‐Luc*, *Myc‐AvrPiz‐t* and *Myc‐AvrPiz‐t (C70A)* were used for co‐transfection into NPB protoplasts. Myc‐Luc was used as the negative control.

*APIP4* encodes a Bowman–Birk‐type trypsin inhibitor (BBI), which is classified as a member of the serine protease inhibitors (Clemente *et al*., 2011). To determine whether APIP4 is a functional inhibitor, we tested its activity in *Escherichia coli* and in rice protoplasts. Trypsin inhibitory ability was determined by end‐point analysis (James *et al*., 2017). Both the APIP4 and the APIP4 mutant, APIP4^K198A ^(Terada *et al*., 1978), which bears a K‐to‐A substitution in the protease‐binding site (Figure S3), were fused with the MBP at its N terminus. The trypsin inhibitor assay showed that increasing amounts of the MBP‐APIP4 fusion protein led to decreasing trypsin activity, indicating it is a functional trypsin inhibitor, which is similar to the positive control, soya bean BBI (SBBI) (James *et al*., 2017) (Figure 2a). However, the mutant MBP‐APIP4^K198A^ showed much lower trypsin inhibitor activity than that of MBP‐APIP4 (Figure 2a), indicating that the protease‐binding site in APIP4 is essential for its trypsin inhibitor activity.

To explore the APIP4 biochemical function *in planta*, we conducted a trypsin inhibitor activity assay for APIP4 and APIP4^K198A^ in rice protoplasts. The *APIP4* and *APIP4^K198A^* fragments were cloned into the pRTVnHA vector under the control of the maize *Ubiquitin* promoter (He *et al*., 2018). Plasmids of *APIP4* and *APIP4^K198A^* fused with a HA tag were transfected into NPB protoplasts, and a *HA‐Luc* construct was used as the negative control. Compared to HA‐Luc, the APIP4 protein in 50 µL of rice protoplasts produced about a 57% reduction in trypsin inhibitor activity (Figure 2b). In contrast, APIP4^K198A^ had about a 28% reduction in activity. The protein levels of APIP4 and APIP4^K198A^ in the rice protoplasts were confirmed by immunoblot analysis (Figure S4a). These results demonstrate that APIP4 functions as a trypsin inhibitor both *in vitro* and *in vivo*.

To test whether AvrPiz‐t affects APIP4 trypsin inhibitor activity *in vitro* and *in vivo*, we purified MBP‐APIP4, His‐fused AvrPiz‐t, and AvrPiz‐t (C70A) (Wang *et al*., 2016), which bears a C‐to‐A substitution and leads to a weak interaction with APIP4 in yeast (Figure S1a). PCR results confirmed the presence of AvrPiz‐t, AvrPiz‐t (C70A) and APIP4 in yeast cells (Figure S1b). A trypsin inhibitor activity assay showed that the difference in the activity of APIP4 in the absence and presence of AvrPiz‐t was substantial (Figure 2c). When AvrPiz‐t was added to the reaction, the trypsin inhibitor activity of APIP4 decreased dramatically, whereas adding AvrPiz‐t (C70A) did not affect the activity substantially. This suggests that AvrPiz‐t inhibits the activity of APIP4, while the mutant AvrPiz‐t (C70A) has lost this inhibitive ability.

Subsequently, we co‐transfected plasmids of *HA‐APIP4* with *Myc‐AvrPiz‐t, Myc‐AvrPiz‐t* (*C70A*) and *Myc‐Luc* to determine whether AvrPiz‐t affects APIP4 activity in rice protoplasts. Compared to the control Myc‐Luc, APIP4 trypsin inhibitor activity was significantly reduced in cells co‐expressing AvrPiz‐t, but was only reduced to about 50% when co‐expressed with AvrPiz‐t (C70A) (Figure 2d). The protein levels of Myc‐AvrPiz‐t, Myc‐AvrPiz‐t (C70A) and HA‐APIP4 in the reactions were confirmed using immunoblots (Figure S4b). Overall, these results demonstrate that AvrPiz‐t suppresses APIP4 trypsin inhibitor activity in *vitro* and in *vivo*.

### APIP4 positively regulates resistance to *M. oryzae*

**Figure 3:**
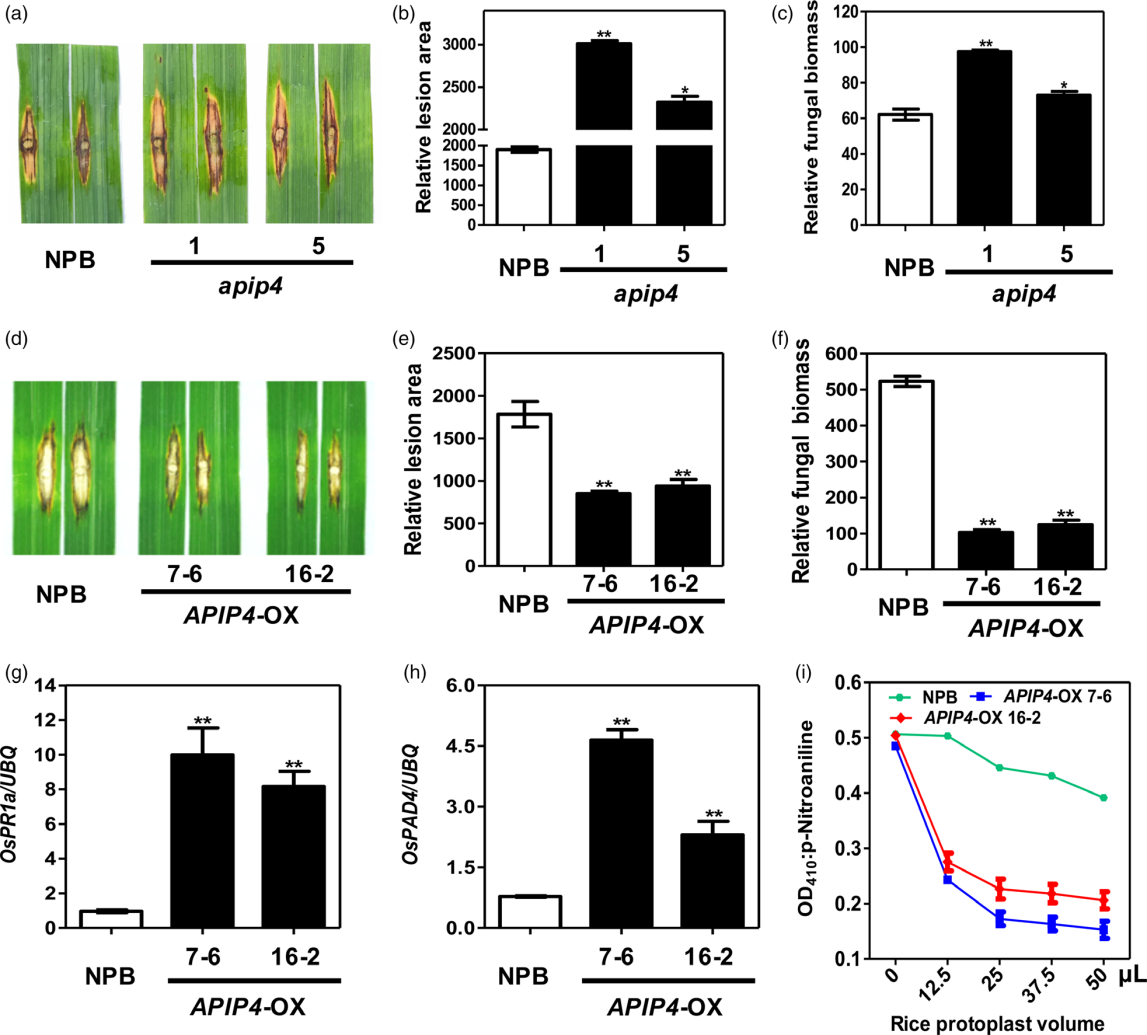
APIP4 positively regulates rice immunity against *M. oryzae*. (a) Punch inoculation of the *apip4* plants. Leaves of eight‐week‐old rice plants were inoculated with the virulent isolate RB22. The leaves were photographed at 14 dpi. (b, c) Relative lesion area (left) and relative fungal biomass (right) were measured 14 dpi. Data shown are means of three replicates. Error bars indicate the SEM (**P < 0.01; *n* = 3). (d) Punch inoculation of *APIP4*‐OX and NPB plants. Leaves of eight‐week‐old rice plants were inoculated with the virulent isolate RB22. The leaves were photographed 14 d post‐inoculation (dpi). (e, f) Relative lesion area (left) and relative fungal biomass (right) were measured at 14 dpi. Data shown are means of three replicates. Error bars indicate the standard error of the mean (SEM) (**P < 0.01; *n* = 3). (g, h) Induction of defence‐related genes *OsPR1a* (g) and *OsPAD4* (h) in the *APIP4*‐OX transgenic and NPB plants by qRT‐PCR. Significance was determined at *P < 0.05 and **P < 0.01 (*n* = 3) with a t‐test. (i) Trypsin inhibitor activity of APIP4 in the *APIP4*‐OX transgenic plants. *In vivo* trypsin inhibitor activity was detected by incubation of an increasing volume of the protoplasts isolated from *APIP4*‐OX transgenic plants with trypsin at 37°C for 20 min. NPB was used as the negative control.

To investigate the role of APIP4 in the immunity of rice to *M. oryzae*, we generated transgenic rice plants that contained insertion and deletion mutations generated by CRISPR/Cas9, or that overexpressed the *APIP4* gene, using *Agrobacterium*‐mediated transformation (Qu *et al*., 2006). To knock out *APIP4,* we designed a guide RNA to target a 20‐nt sequence in the *APIP4* gene for Cas9 cleavage (Figure S5) and cloned it into the pBY02‐OsCas9‐ccdB vector (Zhou *et al*., 2014). After confirming the mutation types of the transgenic lines with PCR and sequencing analyses, we selected two *apip4* T_1_ knockout lines (*apip4*‐1 and 5) for disease phenotype evaluations. *apip4*‐1 carried a 7‐bp deletion in one strand and a 29‐base deletion in the second strand, and *apip‐5* carried a 1‐bp insertion in one strand and a 2‐bp deletion in the second strand (Figure S5). These knockout lines grew normally without any obvious growth penalty. Then, these two *APIP4* knockout lines were punch‐inoculated with the virulent strain RB22 using NPB as the control. The mutant plants showed elevated susceptibility to RB22, with increased lesion area and fungal biomass, compared with NPB (Figure 3a‐c).

To overexpress *APIP4*, we cloned the full‐length gene into the pCXUN vector under the control of the maize *Ubiquitin* promoter (Chen *et al*., 2009). We selected two of the T_2_ homozygous *APIP4* overexpression lines (*APIP4*‐OX 7‐6 and 16‐2) and confirmed their elevated *APIP4* RNA levels by qRT‐PCR, and their protein expression levels by immunoblotting (Figure S6a,b). Similarly, these overexpression lines also grew normally without any obvious phenotype changes. Next, when the *APIP4*‐OX plants were inoculated with the *M. oryzae* virulent isolate RB22, smaller lesions over a smaller area and a decrease in fungal biomass were observed in the overexpression plants compared with the control NPB plants (Figure 3d–f).

The expression levels of defence marker genes such as *OsPR1a* and *OsPAD4* were consistently up‐regulated in the *APIP4*‐OX lines 7‐6 and 16‐2 compared with NPB plants (Figure 3g, h). Thus, the results from the knockout and overexpression lines suggest that *APIP4* functions as a positive regulator in the resistance to *M. oryzae*.

### APIP4 trypsin inhibitor activity is modulated in the *AvrPiz‐t* transgenic rice plants

**Figure 4:**
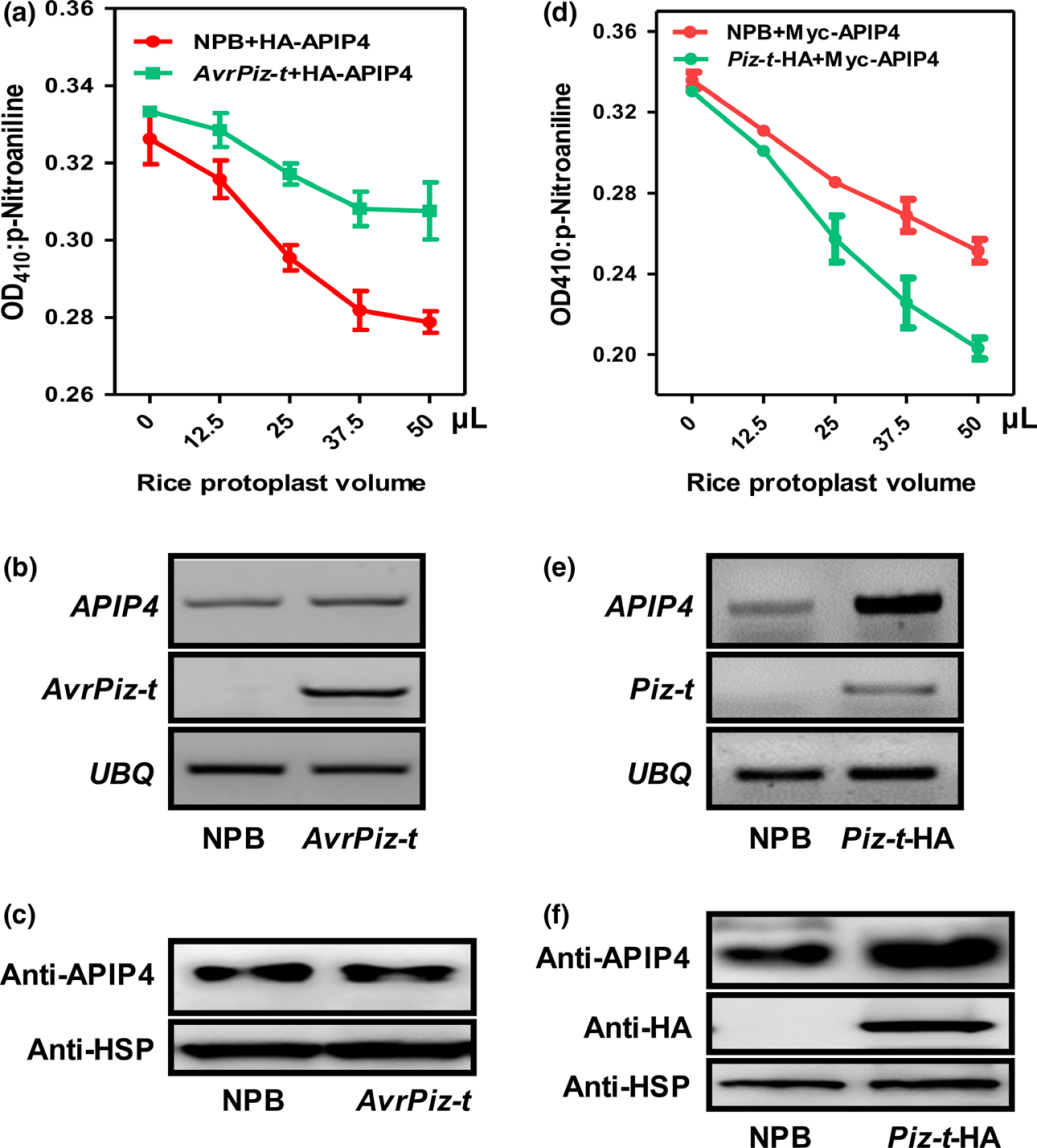
APIP4 trypsin inhibitor activity is differentially modulated in the *AvrPiz‐t* and *Piz‐t*‐HA transgenic plants. (a) APIP4 trypsin inhibitor activity is suppressed in the *AvrPiz‐t* transgenic plants. (b) Transcript levels of *APIP4* were measured by RT‐PCR in two‐week‐old NPB and *AvrPiz‐t* plants. The *Ubiquitin* gene *UBQ* was used as the internal control. (c) APIP4 protein levels were determined in two‐week‐old NPB and *AvrPiz‐t* plants by immunoblotting using the anti‐APIP4 antibody. The HSP protein was used as the internal control. (d) Enhancement of APIP4 trypsin inhibitor activity by Piz‐t. (e) Transcript levels of *APIP4* were measured by RT‐PCR in two‐week‐old NPB and *Piz‐t*‐HA plants. The *Ubiquitin* gene *UBQ* was used as the internal control. (f) APIP4 protein levels were determined in two‐week‐old NPB and *Piz‐t*‐HA plants by immunoblotting using the anti‐APIP4 antibody. The HSP protein was used as the internal control.

To further determine the APIP4 biochemical function in rice, we detected APIP4 trypsin inhibitor activity in the protoplasts of the two *APIP4*‐OX lines (7‐6 and 16‐2) generated above using NPB as the control. The assay revealed that the APIP4 proteins in the two overexpression lines exhibited an increasing ability to inhibit trypsin with increasing amounts of the protoplasts isolated from the two overexpression lines (Figure 3i). To confirm the above results that AvrPiz‐t suppresses APIP4 trypsin inhibitor activity, we attempted to detect APIP4 activity in the *AvrPiz‐t* transgenic plants generated in our previous study (Park *et al*., 2012). The *HA‐APIP4* plasmids were transfected into protoplasts isolated from NPB and the *AvrPiz‐t* transgenic plants. The assay showed that APIP4 activity in the *AvrPiz‐t* transgenic rice protoplasts was significantly reduced compared with that in the NPB protoplasts when equal amounts of rice protoplasts were tested (Figure 4a), confirming that AvrPiz‐t genuinely inhibits APIP4 activity. The protein levels of APIP4 were the same in the NPB and *AvrPiz‐t* rice protoplasts (Figure S7a). To exclude the possibility that AvrPiz‐t affects APIP4 stability, we investigated the *APIP4* transcript and protein levels in the *AvrPiz‐t* transgenic plants and found that AvrPiz‐t did not affect either *APIP4* transcription or protein stability (Figure 4b,c).

### Piz‐t increases APIP4 trypsin inhibitor activity, transcript levels, and protein accumulation

**Figure 5:**
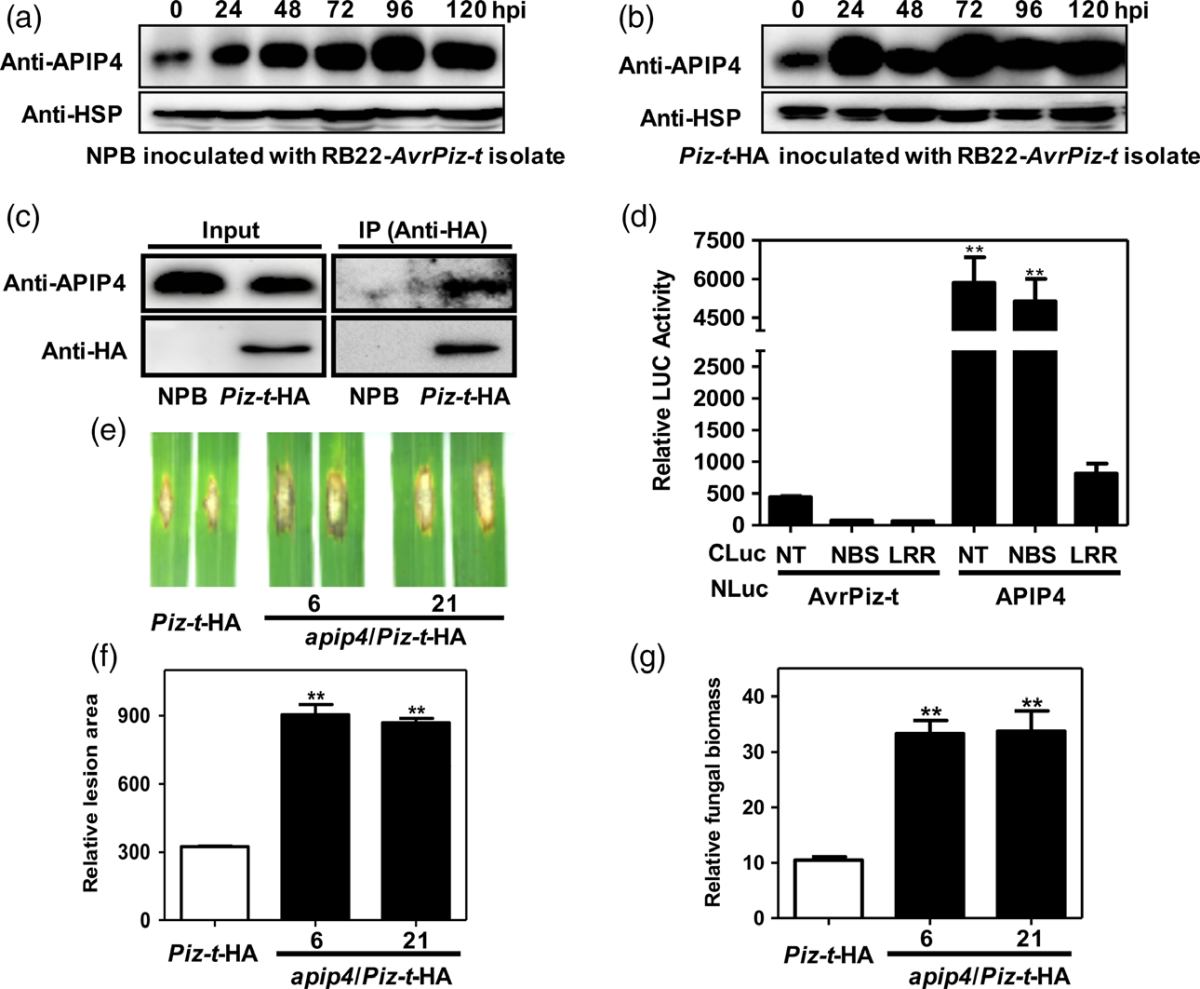
APIP4 interacts with Piz‐t, and knocking out *APIP4* affects Piz‐t immunity to a virulent strain. (a, b) APIP4 protein levels in NPB and *Piz‐t‐*HA plants after inoculation with isolate RB22‐*AvrPiz‐t*. The leaf tissue was harvested at 0, 24, 48, 72, 96 and 120 h after inoculation. APIP4 protein levels were determined by immunoblotting using the anti‐APIP4 antibody. The HSP protein was used as the internal control. (c) Co‐IP assay of Piz‐t and APIP4 in NPB and *Piz‐t*‐HA transgenic plants. Total protein extracted from the NPB and *Piz‐t*‐HA plants was immunoprecipitated with the anti‐HA antibody. Immunoblot analysis was performed using the anti‐APIP4 antibody. As a valid negative control, we used inoculated NPB plants and non‐inoculated Piz‐t‐HA plants to ensure the high concentration of APIP4 protein extracted from NPB. (d) Quantification of the interaction between Piz‐t‐NT/NBS/LRR fragments and APIP4 by the LCI assay. *N. benthamiana* leaves were co‐infiltrated with *Agrobacterium* strains containing APIP4/AvrPiz‐t‐NLuc and CLuc‐Piz‐t‐NT/NBS/LRR. The combinations of the co‐infiltrations are indicated below the panel. AvrPiz‐t‐NLuc was used as the negative control. Error bars indicate the SEM (**P < 0.01; *n* = 3). (e) Punch inoculation of *apip4*/*Piz‐t*‐HA plants. Leaves of eight‐week‐old rice plants were inoculated with the virulent isolate RB22. The leaves were photographed 14 dpi. (f, g) Relative lesion area (left) and relative fungal biomass (right) were measured 14 dpi. Data shown are means of three replications. Error bars indicate the SEM (**P < 0.01; *n* = 3).

Because AvrPiz‐t is indirectly recognized by the cognate resistance protein Piz‐t in rice to induce immunity (Li *et al*., 2009), we hypothesized that APIP4 may be associated with Piz‐t‐mediated resistance. To test this hypothesis, we first tested whether Piz‐t can regulate APIP4 activity. We transfected plasmids of *Myc‐APIP4* into rice protoplasts isolated from *Piz‐t*‐HA and NPB plants and measured APIP4 trypsin inhibitor activity. The assay revealed that APIP4 activity increased more quickly in the *Piz‐t*‐HA rice protoplasts than in the NPB protoplasts when equal amounts of rice protoplasts were tested (Figure 4d). Immunoblot analysis showed that APIP4‐transfected protein levels were the same in NPB and *Piz‐t*‐HA protoplasts (Figure S7b). Next, we determined whether Piz‐t can potentiate *APIP4* transcript and APIP4 protein levels. RNA and protein were isolated from two‐week‐old NPB and *Piz‐t*‐HA plants. RT‐PCR and immunoblot analyses showed that both the transcript and protein levels of APIP4 were constitutively higher in the *Piz‐t*‐HA plants than in the NPB plants (Figure 4e, f). To further investigate whether APIP4 is induced during *M. oryzae* infection, we spray‐inoculated two‐week‐old NPB and *Piz‐t*‐HA plants with the *M. oryzae* virulent isolate RB22 and avirulent isolate RB22‐*AvrPiz‐t,* which was a transformant with the *AvrPiz‐t* gene in the RB22 background. Immunoblot analysis using the proteins isolated from RB22‐*AvrPiz‐t‐*infected plants indicated that more APIP4 accumulated in *Piz‐t*‐HA plants compared with NPB plants. APIP4 rapidly accumulated 24 h after inoculation in *Piz‐t*‐HA plants but only after 48–96 h in NPB plants (Figure 5a, b). This indicated that APIP4 accumulates earlier and at higher levels in the *Piz‐t* background when infected with an *AvrPiz‐t*‐containing strain than when non‐infected. Meanwhile, immunoblot analysis using the protein isolated from the RB22‐infected *Piz‐t*‐HA plants indicated that less APIP4 was accumulated at 24 h after inoculation compared with the avirulent isolate RB22‐*AvrPiz‐t* (Figure S8a and Figure 5b). Taken together, these results suggest that Piz‐t enhances APIP4 trypsin inhibitor activity, transcript and protein accumulation and APIP4 may contribute to *Piz‐t*‐mediated resistance. To further determine whether APIP4 protein accumulation in NPB and *Piz‐t*‐HA plants after inoculation with RB22‐*AvrPiz‐t* depends on the transcriptional reprogramming, we performed qRT‐PCR with the same samples used in Figure 5a,b and found that *APIP4* transcription levels were only induced at the early stage, implying that APIP4 protein accumulation does not depend on the transcriptional reprogramming (Figure S8b).

### APIP4 interacts with Piz‐t, and knocking out *APIP4* affects Piz‐t immunity to a virulent *M. oryzae* strain

To further determine the relationship between APIP4 and Piz‐t, we performed a Co‐IP assay using the protein isolated from NPB and *Piz‐t*‐HA transgenic plants with the anti‐HA and anti‐APIP4 antibodies. The immunoblot analyses showed that the expected APIP4 band was detected using the endogenous APIP4 antibody in the *Piz‐t*‐HA immunoprecipitated proteins but not in NPB plants (Figure 5c). Furthermore, we used split‐luciferase image (LCI) analysis to determine which Piz‐t domains interact with APIP4 in *Nicotiana benthamiana* (Chen *et al*., 2008). The truncated *Piz‐t* fragments, NT, NBS, and LRR, were fused to the C terminus of the *LUC* gene in the pCAMBIA‐CLuc vector for the generation of CLuc‐NT, NBS and LRR constructs. The *APIP4* gene was fused to the N terminus of *LUC* in the pCAMBIA‐NLuc vector (APIP4‐NLuc). AvrPiz‐t fused to the N terminus of *LUC* in the pCAMBIA‐NLuc vector (AvrPiz‐t‐NLuc) was used as the negative control. The LCI analysis showed that co‐expression of APIP4‐NLuc with CLuc‐NT or CLuc‐NBS produced strong LUC activity but not with CLuc‐LRR (Figure 5d), indicating that both the NT and NBS fragments of Piz‐t interact with APIP4. Strong fluorescence was also detected with a low‐light imaging system in *N. benthamiana* leaves in which the NT and NBS fragments of Piz‐t and APIP4 were co‐infiltrated, but not the LRR domain (Figure S9a). These results demonstrate that APIP4 interacts with the NT and NBS domains of Piz‐t *in vitro* and *in vivo*.

To examine whether Piz‐t‐mediated rice blast resistance depends on APIP4, we generated *APIP4* knockout lines in the *Piz‐t‐HA* background (*apip4*/*Piz‐t*‐HA) using the CRISPR/Cas9 method. We selected two knockout lines (*apip4*/*Piz‐t*‐HA 6 and 21) for phenotype evaluations. The mutations in the two transgenic lines were confirmed by PCR and sequencing analyses. We found that *apip4*/*Piz‐t*‐HA 6 carries a 2‐bp deletion in one strand and a 1‐bp insertion in the second strand and that *apip4*/*Piz‐t*‐HA 21 is a homozygous line carrying a 1‐bp insertion (Figure S9b), both of which truncated the *APIP4* open reading frame. When the *apip4*/*Piz‐t*‐HA plants were challenged with the avirulent isolate RO1‐1, we found that the transgenic plants had the similar resistance level as the *Piz‐t*‐HA plants (Figure S9c). However, when we inoculated the *apip4*/*Piz‐t*‐HA plants with the virulent isolate RB22, the plants showed enhanced susceptibility to *M. oryzae* with a greater lesion area and more fungal biomass compared with the *Piz‐t*‐HA plants (Figure 5e‐g). These results imply that knocking out *APIP4* does not affect Piz‐t‐mediated resistance against avirulent isolates, but could decrease the resistance against virulent isolates.

## Discussion

### The *M. oryzae* cytoplasmic effector directly binds and interferes host protease inhibitor activity

Plant pathogens secrete both protease inhibitors and proteases into host cells for immune suppression. Many protease inhibitors act as effectors to target the host proteases and thus subvert plant immunity (Shabab *et al*., 2008). How host protease inhibitors inhibit pathogen proteases for defence is not fully understood. A recent study showed that the *P. sojae* apoplastic effectors PsXEG1 and PsXLP1 target the soya bean apoplastic protease inhibitor GmGIP1 (Ma *et al*., 2016), suggesting a ‘bodyguard’ strategy in which the paralogous decoy PsXLP1 protects the virulence factor PsXEG1 in the extracellular space (Paulus *et al*., 2017).

In this study, our results revealed that the cytoplasmic effector AvrPiz‐t targets a host trypsin inhibitor, APIP4. We demonstrated that APIP4 is a functional BBI, which exhibits the trypsin inhibitor activity *in vitro* and *in vivo*. Furthermore, AvrPiz‐t can specifically target and inhibit APIP4 trypsin inhibitor activity *in vitro* and *in vivo*. Notably, the *APIP4* overexpression plants show enhanced trypsin inhibitor activity, while the APIP4 trypsin inhibitor activity is significantly reduced in the *AvrPiz‐t* transgenic rice protoplasts. Although the protease from *M. oryzae* that targets APIP4, how AvrPiz‐t affects APIP4 trypsin inhibitor activity and whether the trypsin inhibitor activity of APIP4 is indeed involved in rice immunity are still unclear, our study suggests that *M. oryzae* effectors can target host protease inhibitors in the intracellular space.

### The rice BBI APIP4 confers enhanced resistance against the destructive rice pathogen *M. oryzae*

Soya bean BBIs confer antitumour, anti‐inflammation and antiviral activities in mammals (Kennedy, 1998; Safavi and Rostami, 2012). For example, a soya bean BBI can suppress MMP‐2 and MMP‐9 enzyme activity to inhibit the growth of the two adenocarcinoma cells AGS and HT29 (Fereidunian *et al*., 2014). And the soya bean BBI can block HIV entry into macrophages (Ma *et al*., 2018). BBI homologs were also identified as antimicrobial proteins in wheat, maize and rice (Chen *et al*., 1999; Chilosi *et al*., 2000; Qu *et al*., 2003). However, the molecular mechanisms of BBI‐mediated immunity are largely unknown. In this study, we demonstrated that the rice BBI APIP4 plays a positive role in rice immunity. After *M. oryzae* infection, the level of *APIP4* protein greatly increased. Overexpression of *APIP4* in transgenic rice consistently enhanced rice blast resistance, while knocking out *APIP4* increased susceptibility to this pathogen. The positive role of APIP4 in resistance can be explained by the up‐regulation of defence‐related marker genes in the *APIP4* overexpression plants. However, how APIP4 interacts with other proteins to trigger defence responses remains unknown.

### The function of the rice BBI APIP4 is reinforced by the NLR protein Piz‐t

In this study, we found that APIP4 interacts with rice blast NLR Piz‐t *in vivo* and Piz‐t increases APIP4 trypsin inhibitor activity when they are co‐expressed. In addition, both the *APIP4* transcript level and APIP4 protein accumulation are induced in the *Piz‐t*‐expressing plants. This is similar to the relationship between the transcription factor WRKY45 and the rice blast NLR protein Pb1 (Inoue *et al*., 2013). WRKY45 interacts with Pb1, and transient expression of Pb1 in rice protoplasts stabilized WRKY45 accumulation and enhanced its transactivation activity. Genetic analysis demonstrated that Pb1‐mediated blast resistance is dependent on WRKY45 (Inoue *et al*., 2013). However, different from the relationship between Pb1 and WRKY45, knocking out *APIP4* in the *Piz‐t*‐HA background does not affect *Piz‐t* resistance to an avirulent strain, while it enhances susceptibility to a virulent strain.

Our previous studies showed that APIP5 positively regulates Piz‐t protein accumulation and APIP10 negatively regulates Piz‐t accumulation (Park *et al*., 2016; Wang *et al*., 2016). Thus, it is possible that other Piz‐t‐associated proteins, such as APIP5 and APIP10, or unknown proteins, have redundant functions in the regulation of *Piz‐t*‐mediated resistance when *APIP4* is knocked out (Park *et al*., 2016; Wang *et al*., 2016). Actually, the phylogenetic tree analysis indicated that APIP4 has 11 homologous members in rice (Figure S10a). Our Y2H assay showed that the APIP4 closest homolog OsBBTI5 also interacts with AvrPiz‐t (Figure S10b). Furthermore, the LCI assay indicated that OsBBTI5 can interact with APIP4 (Figure S10c), implying that other BBTIs may be also involved in the Piz‐t‐mediated resistance. It is also possible that Piz‐t recognizes another unknown *M. oryzae* effector that leads to immediate resistance and APIP4 is required for the resistance. Therefore, further studies are necessary to better understand how Piz‐t modulates APIP4 in rice innate immunity, and the role of APIP4 in the Piz‐t background against virulent strains.

### A working model for the relationship among the AvrPiz‐t, APIP4 and Piz‐t proteins during *M. oryzae* infection

**Figure 6:**
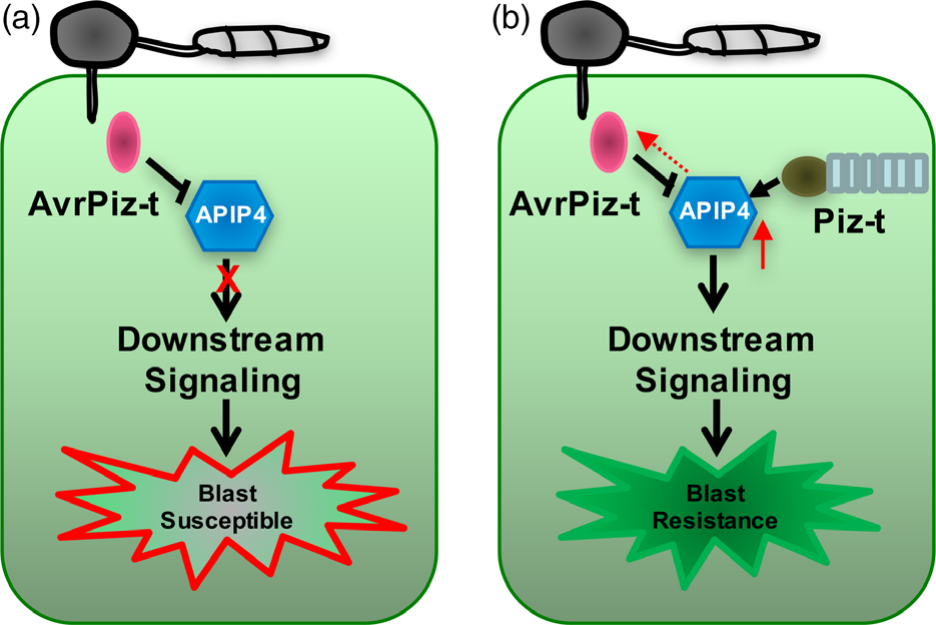
A working model for the interaction among AvrPiz‐t, APIP4 and Piz‐t. (a) Upon *M. oryzae* infection, the effector AvrPiz‐t is secreted into the cytoplasm to interact with APIP4 and inhibit its trypsin inhibitor activity, reducing APIP4‐mediated basal resistance and resulting in plant susceptibility. (b) In the *Piz‐t*‐containing plants, Piz‐t directly binds APIP4, which may lead to the enhancement of APIP4 protein level (red solid arrow) and trypsin inhibitor activity to attenuate AvrPiz‐t interference (red dashed arrow towards AvrPiz‐t) and trigger downstream immune responses.

Based on our results, we propose a working model to illustrate the relationship among AvrPiz‐t, APIP4 and Piz‐t (Figure 6). Upon *M. oryzae* infection, *M. oryzae* secretes the effector AvrPiz‐t into the cytoplasm where it interacts with APIP4, inhibiting APIP4 activity to impair plant immunity (Figure 6a). In the *Piz‐t*‐expressing plants, Piz‐t interacts with APIP4, and enhances its transcript and protein levels, and inhibitor activity, possibly reducing the attenuation by AvrPiz‐t (Figure 6b).

Our work demonstrates a novel mechanism in which a protease inhibitor from a host is targeted by the effector from a pathogen, and also maybe act as a downstream component of the host NLR protein. Overall, our study reveals a novel host–microbe relationship in which a BBI inhibitor can be modulated by a pathogen cytoplasmic effector, but an NLR receptor can reinforce its inhibitor activity for plant immunity.

## Experimental procedures

### Plant materials and growth conditions

Rice (*Oryza sativa* L.) lines, including NPB and the *Piz‐t* transgenic line in NPB (NPB‐*Piz‐t*). Rice transgenic materials for phenotype analysis, including CRISPR or overexpression plants, are from T2 generation. Rice plants are grown in pots and kept in a growth chamber at 26°C and 70% relative humidity with the cycle of 12‐h light/12‐h dark.

### Punch inoculation of rice leaves

Spore concentration in the suspension was adjusted to 5 × 10^5^ conidia/mL before punch inoculation. The rice leaves were lightly punched with a mouse ear punch, and a 10 μL volume of spore suspension was dropped onto the punched sites of leaves. The spore suspension was secured by sealing with Scotch tape on both sides. The relative fungal biomass was measured with DNA‐based quantitative PCR (qPCR) using the threshold cycle value (CT) of the *M. oryzae MoPot2* gene against the CT value of the rice *ubiquitin* gene (Park *et al*., 2012). All inoculation experiments were repeated three times independently.

### Rice protoplast isolation and transfection

Ten‐day‐old etiolated rice seedlings grown on 1⁄2 MS medium in the dark were used for protoplast isolation (He *et al*., 2016). The sheath and stem of seedlings were cut into 0.5‐mm strips and soaked in cell wall digestion buffer (1.5% cellulase RS, 0.75% macerozyme R‐10, 0.6 m mannitol, 10 mm MES, 10 mm CaCl_2_, 0.1 % BSA, pH 5.7) for 5 h in the dark with gentle shaking (60–80 rpm). After digestion, strips were washed with W5 solution (154 mm NaCl, 125 mm CaCl_2_, 5 mm KCl and 2 mm MES, 0.5 % glucose, pH 5.7) and filtered with 40‐mm nylon mesh. Protoplasts were collected by centrifugation at 1000 *g* for 3 min and washed with W5 three times, then suspended in W5 solution to a concentration of 2 × 10^6^ cells per mL.

Protoplast transfection was performed using the PEG‐mediated method. About 5–10 µg plasmid DNA was mixed with 100 µL protoplasts. An equal volume of PEG solution [40 % (W/V) PEG 4000 (Sigma‐Aldrich, Louis, MO), 0.2 m mannitol and 0.1 m CaCl_2_] was added and mixed by inverting tubes gently. The mixture was incubated at room temperature for 20 min. Then, a double volume of W5 solution was added to the mixture and mixed by inversion. Protoplasts were collected 36 h after incubation by centrifugation, and the pellet was suspended in W5 solution (He *et al*., 2016).

### Trypsin inhibition activity measurement *in vitro* and *in vivo*

Trypsin inhibition activity was determined as previously described with slight modifications (James *et al*., 2017). SBBI from soya bean was used as a positive control (Sigma‐Aldrich). In the reaction, increasing amounts of proteins or increasing volumes of the protoplast lysate were added to 50 μL of 20 μg/mL bovine pancreatic trypsin (Sigma‐Aldrich) dissolved in 50 mm Tris‐Cl and 20 mm CaCl_2_, pH 8.0, and incubated for 15 min at 37°C. Trypsin activity was determined by adding 125 μL of 1 mm N‐α‐benzoyl‐DL‐arginine‐p‐nitroanilide (BAPNA) substrate (Sigma‐Aldrich) dissolved in 50 mm Tris‐HCl, 20 mm CaCl_2_, pH 8.0, and 1% (v/v) dimethyl sulphoxide, and incubated at 37°C for 20 min. The reaction was stopped with the addition of 25 μL of 30% (v/v) acetic acid. Total reaction volume was 0.25 mL. The absorbance was measured at 410 nm.

### *In vitro* pull‐down assay

The GST fusion proteins, GST: APIP4‐C, and the MBP fusion proteins, MBP:AvrPiz‐t and MBP:APIP4, were expressed in *E. coli*. After cell lysis, GST and GST:APIP4‐C were mixed with an equal amount of the MBP fusion proteins, and then, the mixture was added to 1ml binding buffer (50 mm Tris at pH 8.0, 120 mm NaCl, 1 mm DTT, 0.5 % NP‐40, 1 mm PMSF). The mixture was incubated at room temperature for 1 h with gentle shaking, and then, 20 μL glutathione‐Sepharose beads (GE Healthcare) were added, followed by incubation at room temperature for 1 h (Shi et al., 2018). The beads were then washed five times with washing buffer (50 mm Tris at pH8.0, 500 mm NaCl, 1 mm DTT, 0.5% NP‐40, 1 mm PMSF). Immunoblots were used for detection of fusion proteins with the anti‐MBP and anti‐GST antibodies.

### *In vivo* Co‐IP assay

For Co‐IP experiments using rice protoplasts, total protein was extracted with native extraction buffer [50 mm Tris‐MES at pH 8.0, 0.5 m sucrose, 1 mm MgCl_2_, 10 mm EDTA, 5 mm DTT and protease inhibitor cocktail (Roche, Mannheim, Germany)]. For anti‐HA Co‐IP, the extracted proteins were pre‐rinsed with 30 μL protein A agarose for 1 h. The pre‐rinsed proteins were incubated with 2 μg anti‐HA antibody and protein A agarose for 4 h. The agarose was washed five times with PBST buffer. The bound proteins were boiled with SDS loading buffer for 5 min in water and detected by the anti‐HA (Roche) and anti‐GFP (Roche) antibodies.

### Yeast two‐hybrid (Y2H) assay

The matchmaker GAL4 two‐hybrid system (Invitrogen, Carlsbad, CA) was used for Y2H assay. The coding regions without the signal peptide of AvrPiz‐t, AvrPiz‐t (C70A), AvrPi9 and AvrPii were cloned into the pDBleu vector. The *APIP4‐C* (containing the full intracellular domain) and OsBBTI5‐C fragments were cloned into the pPC86 vector. Constructs were co‐transformed into the yeast strain Mav203 (Wang *et al*., 2016). Positive clones were selected on medium without Leu‐Trp‐His but with 35mM 3AT.

### *In vivo* split‐luciferase assay

APIP4 and AvrPiz‐t were fused to the N terminus of LUC in the pCAMBIA‐NLuc vector, and Piz‐t‐NT/NBS/LRR was fused to the C terminus of LUC in the pCAMBIA‐CLuc vector (Chen *et al*., 2008). *A. tumefaciens* strain EHA105 containing the indicated constructs was infiltrated into 5‐ to 6‐week‐old *N. benthamiana* leaves. Three leaf discs were taken after 2 d, and incubated with 200 μL of 100 mm luciferin in a 96‐well plate, and luminescence was measured with the GLOMAX 96 microplate luminometer (Promega, Sunnyvale, CA). Each experiment was performed at least three times.

### Rice protein extraction and immunoblotting

Total protein was extracted from the wild‐type and transgenic rice seedlings with protein denaturing buffer (50 mm Tris‐HCl, pH 7.5, 150 mm NaCl, 0.1 % NP‐40, 4 m urea and protease inhibitor cocktail) or native buffer (50 mm Tris‐MES at pH 8.0, 0.5 m sucrose, 1 mm MgCl_2_, 10 mm EDTA, 5 mm DTT and protease inhibitor cocktail). Supernatants were collected twice by centrifugation at 15,000 g for 15 min. Total protein was separated in SDS‐PAGE and detected by immunoblotting with the antibodies indicated.

### Accession number

The accession number of the *APIP4* gene reported in this paper is LOC_Os01g03340.

## Conflict of interest

The authors declare no conflicts of interest.

## Author contributions

C.Z., H.F., X.S., R.W. and F.H. performed the experiments; Y.N, G‐L.W. and C.Z. designed the experiments; Y.N., G‐L.W. and C.Z. analysed the data; C.Z. wrote the paper. All authors have discussed the results, have commented on the manuscript and have read and approved the final manuscript.

## Supporting information


**Figure S1.** AvrPiz‐t specifically interacts with APIP4 in yeast.
**Figure S2.** APIP4 subcellular localization and its co‐localization with AvrPiz‐t in rice protoplasts.
**Figure S3.** Mutation sites in APIP4.
**Figure S4.** Protein expression level detection in rice protoplasts.
**Figure S5.** The genome editing types of the two *apip4* mutant lines in the NPB background.
**Figure S6.** Genotype identification in the *APIP4*‐OX transgenic plants.
**Figure S7.** Detection of APIP4 protein levels in the protoplasts of *AvrPiz‐t* and *Piz‐t* plants.
**Figure S8.** Detection of APIP4 protein and transcript levels in NPB and *Piz‐t*‐HA plants after inoculation.
**Figure S9.** The Piz‐t interacts with APIP4, but APIP4 doesn’t affect Piz‐t mediated resistance against avirulent isolate.
**Figure S10.** The phylogenetic tree analysis of *APIP4* and its homolog members in rice.
