# Supplementary material for: A fungal effector and a rice NLR protein have antagonistic effects on a Bowman–Birk trypsin inhibitor
Source: Plant Biotechnol J. 2020 Jul 4;18(11):2354–63. doi: 10.1111/pbi.13400 (PMC7589341; doi:10.1111/pbi.13400)
Supplement: Supplementary file 1 — Figure S1. AvrPiz‐t specifically interacts with APIP4 in yeast. Figure S2. APIP4 subcellular localization and its co‐localization with AvrPiz‐t in rice protoplasts. Figure S3. Mutation sites in APIP4. Figure S4. Protein expression level detection in rice protoplasts. Figure S5. The genome editing types of the two apip4 mutant lines in the NPB background. Figure S6. Genotype identification in the APIP4‐OX transgenic plants. Figure S7. Detection of APIP4 protein levels in the protoplasts of AvrPiz‐t and Piz‐t plants. Figure S8. Detection of APIP4 protein and transcript levels in NPB and Piz‐t‐HA plants after inoculation. Figure S9. The Piz‐t interacts with APIP4, but APIP4 doesn’t affect Piz‐t mediated resistance against avirulent isolate. Figure S10. The phylogenetic tree analysis of APIP4 and its homolog members in rice. [file PBI-18-2354-s001.ppt]

## Slide 1
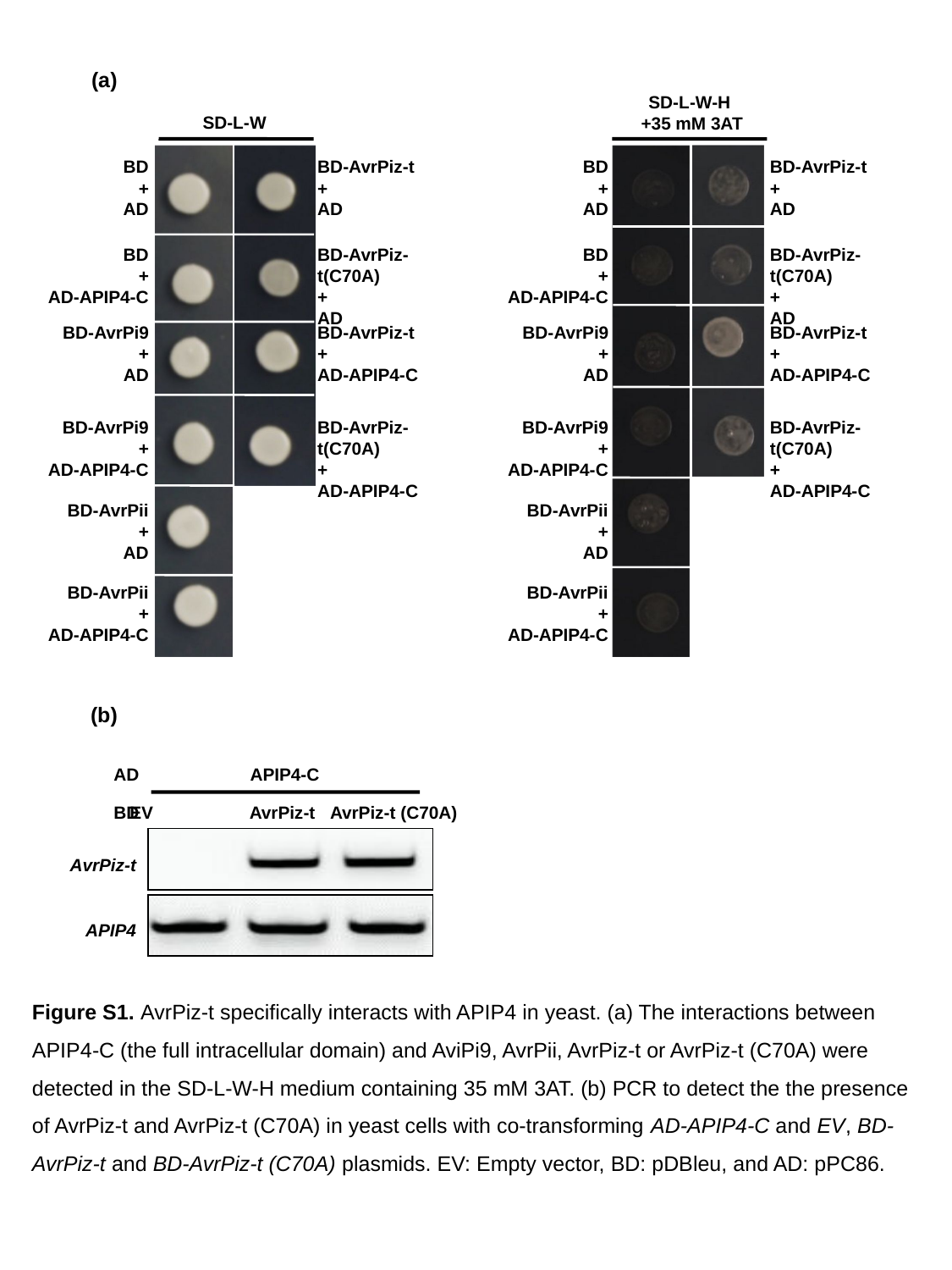

(a)
 SD-L-W-H
 +35 mM 3AT
SD-L-W
BD
+
AD
BD-AvrPiz-t
+
AD
BD
+
AD
BD-AvrPiz-t
+
AD
BD
+
AD-APIP4-C
BD-AvrPiz-t(C70A)
+
AD
BD
+
AD-APIP4-C
BD-AvrPiz-t(C70A)
+
AD
BD-AvrPi9
+
AD
BD-AvrPiz-t
+
AD-APIP4-C
BD-AvrPi9
+
AD
BD-AvrPiz-t
+
AD-APIP4-C
BD-AvrPi9
+
AD-APIP4-C
BD-AvrPiz-t(C70A)
+
AD-APIP4-C
BD-AvrPi9
+
AD-APIP4-C
BD-AvrPiz-t(C70A)
+
AD-APIP4-C
BD-AvrPii
+
AD
BD-AvrPii
+
AD
BD-AvrPii
+
AD-APIP4-C
BD-AvrPii
+
AD-APIP4-C
(b)
AD
APIP4-C
BD
EV
AvrPiz-t
AvrPiz-t (C70A)
AvrPiz-t
APIP4
Figure S1. AvrPiz-t specifically interacts with APIP4 in yeast. (a) The interactions between APIP4-C (the full intracellular domain) and AviPi9, AvrPii, AvrPiz-t or AvrPiz-t (C70A) were detected in the SD-L-W-H medium containing 35 mM 3AT. (b) PCR to detect the the presence of AvrPiz-t and AvrPiz-t (C70A) in yeast cells with co-transforming AD-APIP4-C and EV, BD-AvrPiz-t and BD-AvrPiz-t (C70A) plasmids. EV: Empty vector, BD: pDBleu, and AD: pPC86.

## Slide 2
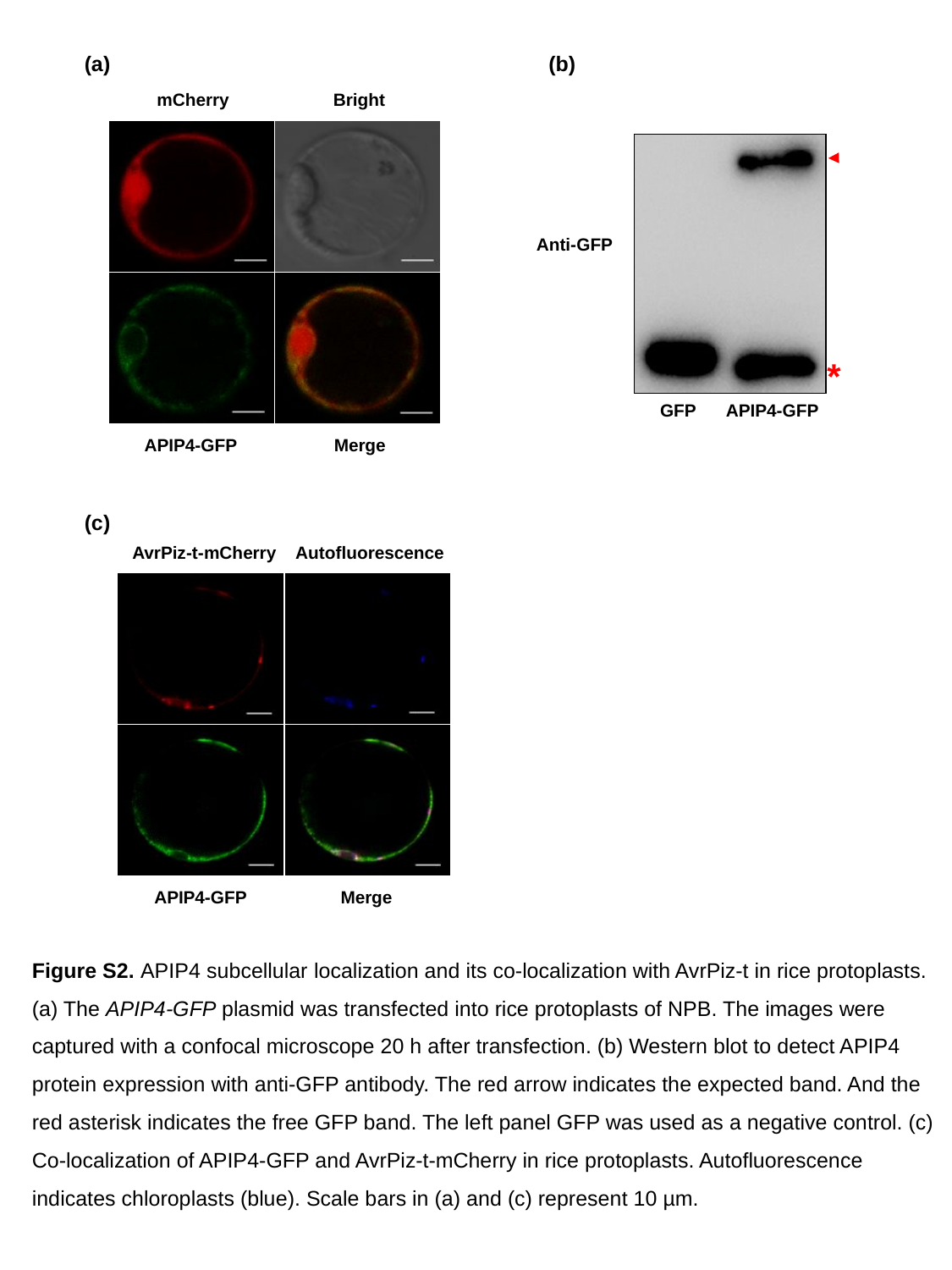

(a)
(b)
mCherry
Bright
Anti-GFP
*
GFP
APIP4-GFP
APIP4-GFP
Merge
(c)
AvrPiz-t-mCherry
Autofluorescence
APIP4-GFP
Merge
Figure S2. APIP4 subcellular localization and its co-localization with AvrPiz-t in rice protoplasts. (a) The APIP4-GFP plasmid was transfected into rice protoplasts of NPB. The images were captured with a confocal microscope 20 h after transfection. (b) Western blot to detect APIP4 protein expression with anti-GFP antibody. The red arrow indicates the expected band. And the red asterisk indicates the free GFP band. The left panel GFP was used as a negative control. (c) Co-localization of APIP4-GFP and AvrPiz-t-mCherry in rice protoplasts. Autofluorescence indicates chloroplasts (blue). Scale bars in (a) and (c) represent 10 µm.

## Slide 3
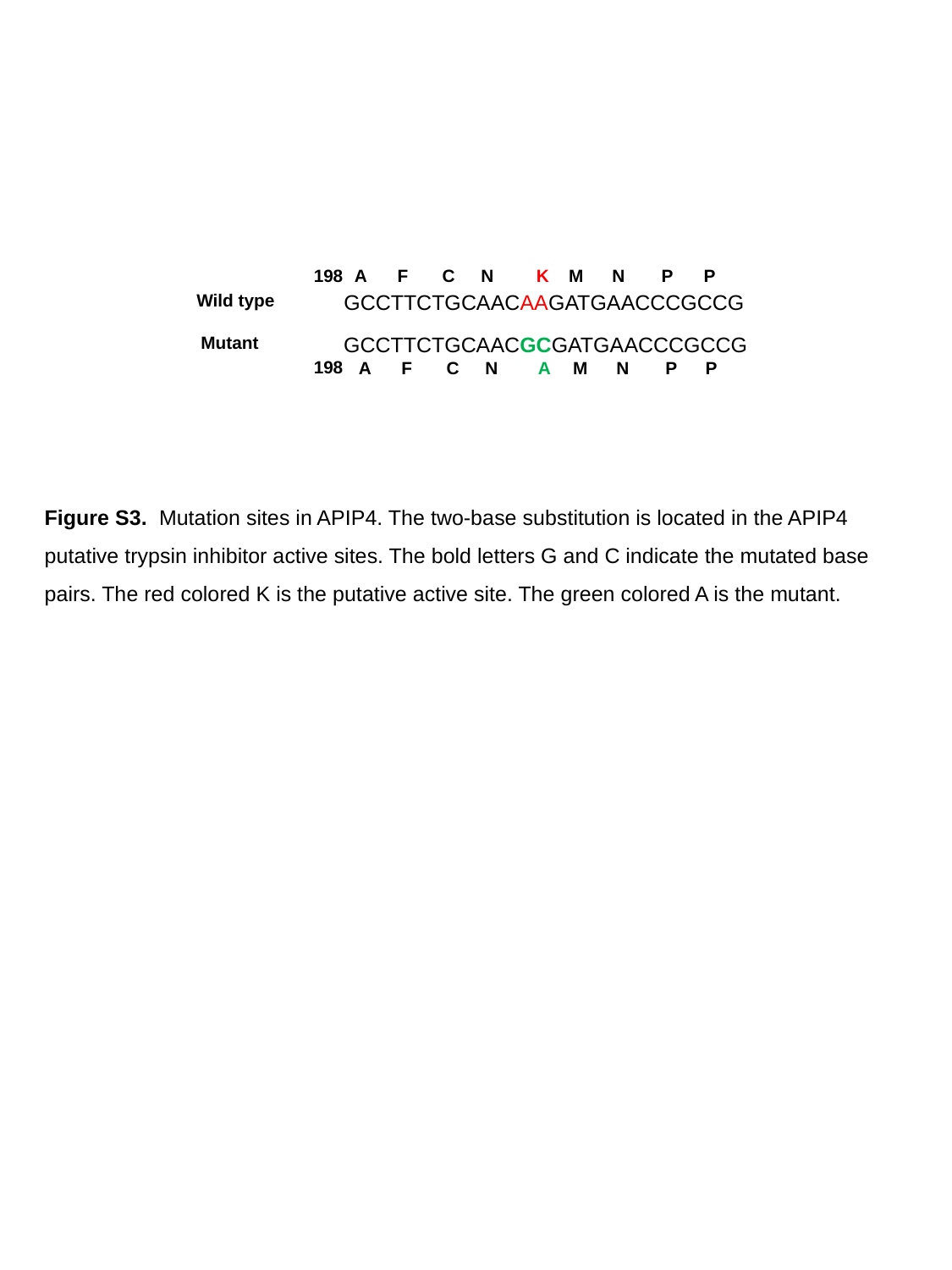

198
A
F
C
N
 K
M
N
P
P
GCCTTCTGCAACAAGATGAACCCGCCG
Wild type
Mutant
GCCTTCTGCAACGCGATGAACCCGCCG
198
A
F
C
N
 A
M
N
P
P
Figure S3. Mutation sites in APIP4. The two-base substitution is located in the APIP4 putative trypsin inhibitor active sites. The bold letters G and C indicate the mutated base pairs. The red colored K is the putative active site. The green colored A is the mutant.

## Slide 4
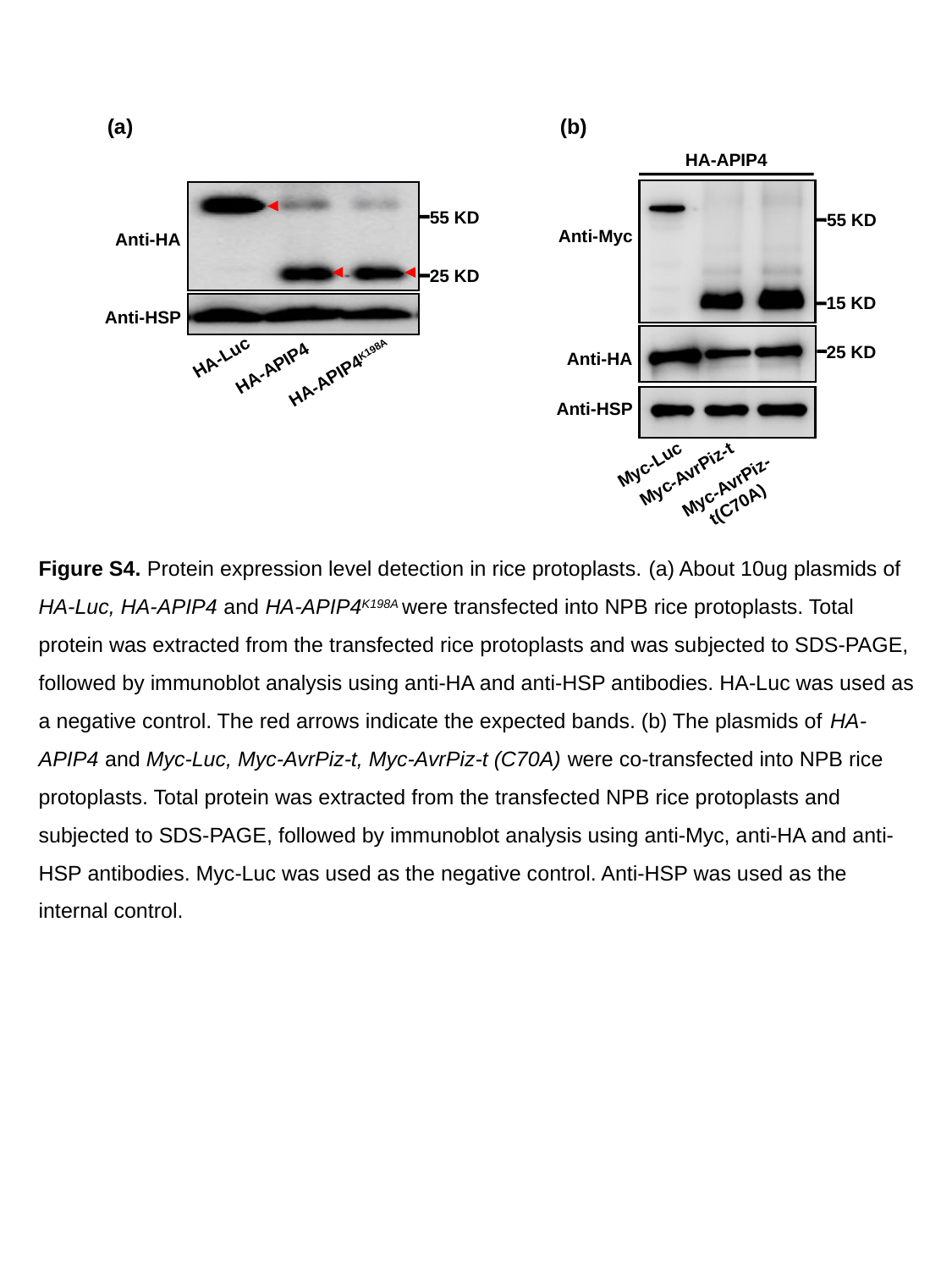

(a)
(b)
HA-APIP4
55 KD
55 KD
Anti-Myc
Anti-HA
25 KD
15 KD
Anti-HSP
25 KD
HA-Luc
Anti-HA
HA-APIP4
HA-APIP4K198A
Anti-HSP
Myc-Luc
Myc-AvrPiz-t
Myc-AvrPiz-t(C70A)
Figure S4. Protein expression level detection in rice protoplasts. (a) About 10ug plasmids of HA-Luc, HA-APIP4 and HA-APIP4K198A were transfected into NPB rice protoplasts. Total protein was extracted from the transfected rice protoplasts and was subjected to SDS-PAGE, followed by immunoblot analysis using anti-HA and anti-HSP antibodies. HA-Luc was used as a negative control. The red arrows indicate the expected bands. (b) The plasmids of HA-APIP4 and Myc-Luc, Myc-AvrPiz-t, Myc-AvrPiz-t (C70A) were co-transfected into NPB rice protoplasts. Total protein was extracted from the transfected NPB rice protoplasts and subjected to SDS-PAGE, followed by immunoblot analysis using anti-Myc, anti-HA and anti-HSP antibodies. Myc-Luc was used as the negative control. Anti-HSP was used as the internal control.

## Slide 5
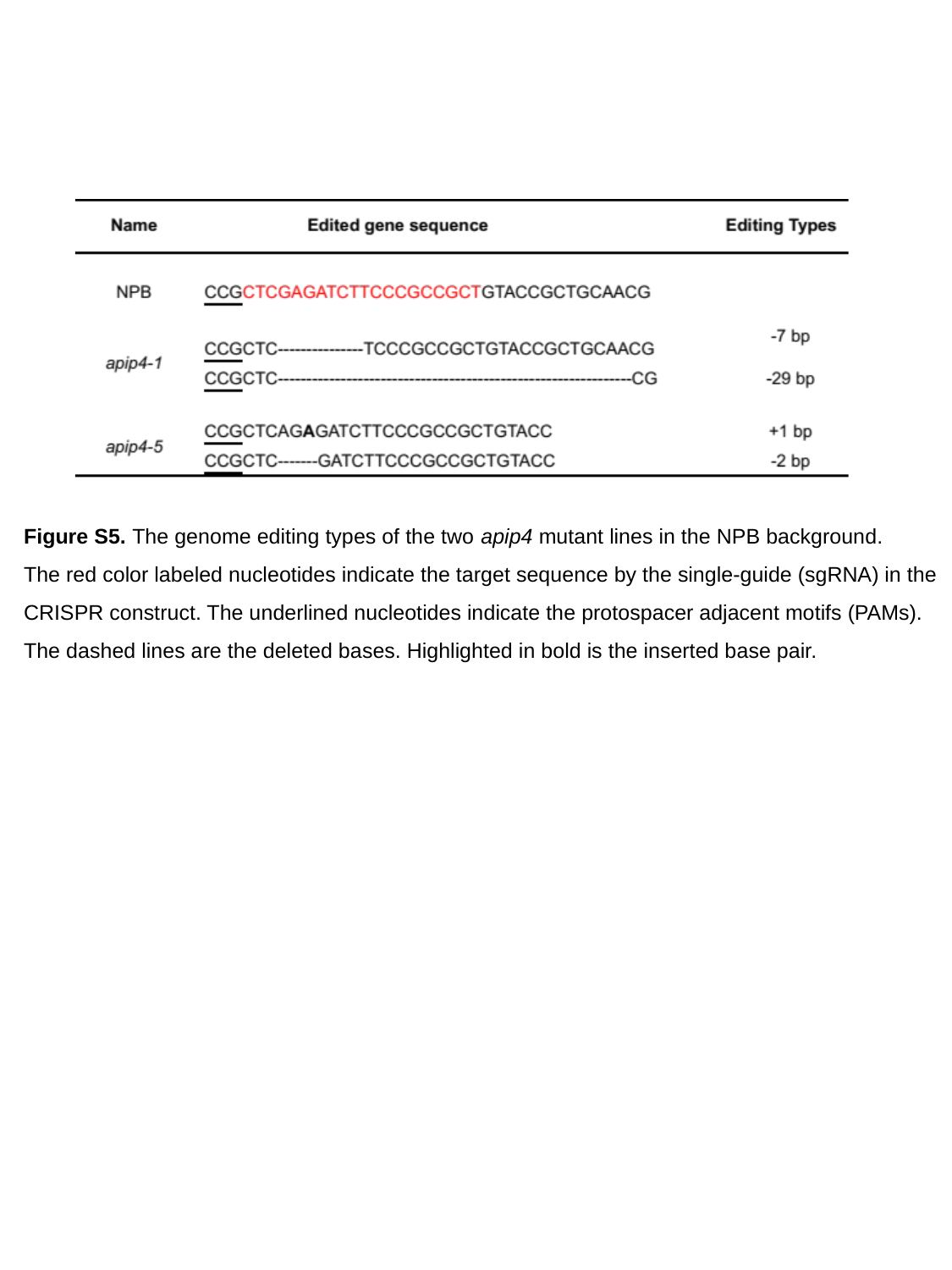

Figure S5. The genome editing types of the two apip4 mutant lines in the NPB background.
The red color labeled nucleotides indicate the target sequence by the single-guide (sgRNA) in the CRISPR construct. The underlined nucleotides indicate the protospacer adjacent motifs (PAMs). The dashed lines are the deleted bases. Highlighted in bold is the inserted base pair.

## Slide 6
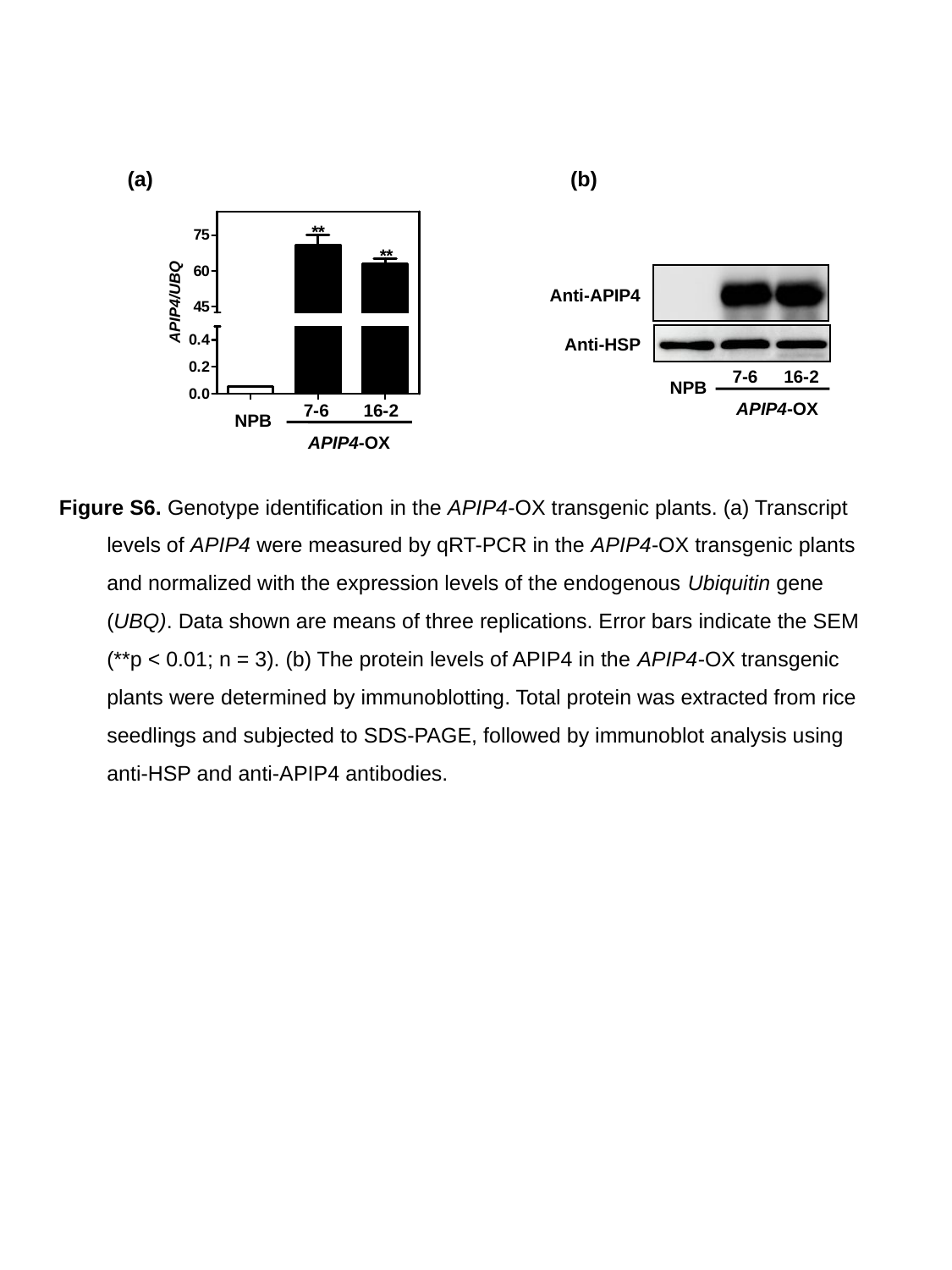

(a)
(b)
Anti-APIP4
Anti-HSP
7-6
16-2
NPB
APIP4-OX
7-6
16-2
NPB
APIP4-OX
Figure S6. Genotype identification in the APIP4-OX transgenic plants. (a) Transcript levels of APIP4 were measured by qRT-PCR in the APIP4-OX transgenic plants and normalized with the expression levels of the endogenous Ubiquitin gene (UBQ). Data shown are means of three replications. Error bars indicate the SEM (**p < 0.01; n = 3). (b) The protein levels of APIP4 in the APIP4-OX transgenic plants were determined by immunoblotting. Total protein was extracted from rice seedlings and subjected to SDS-PAGE, followed by immunoblot analysis using anti-HSP and anti-APIP4 antibodies.

## Slide 7
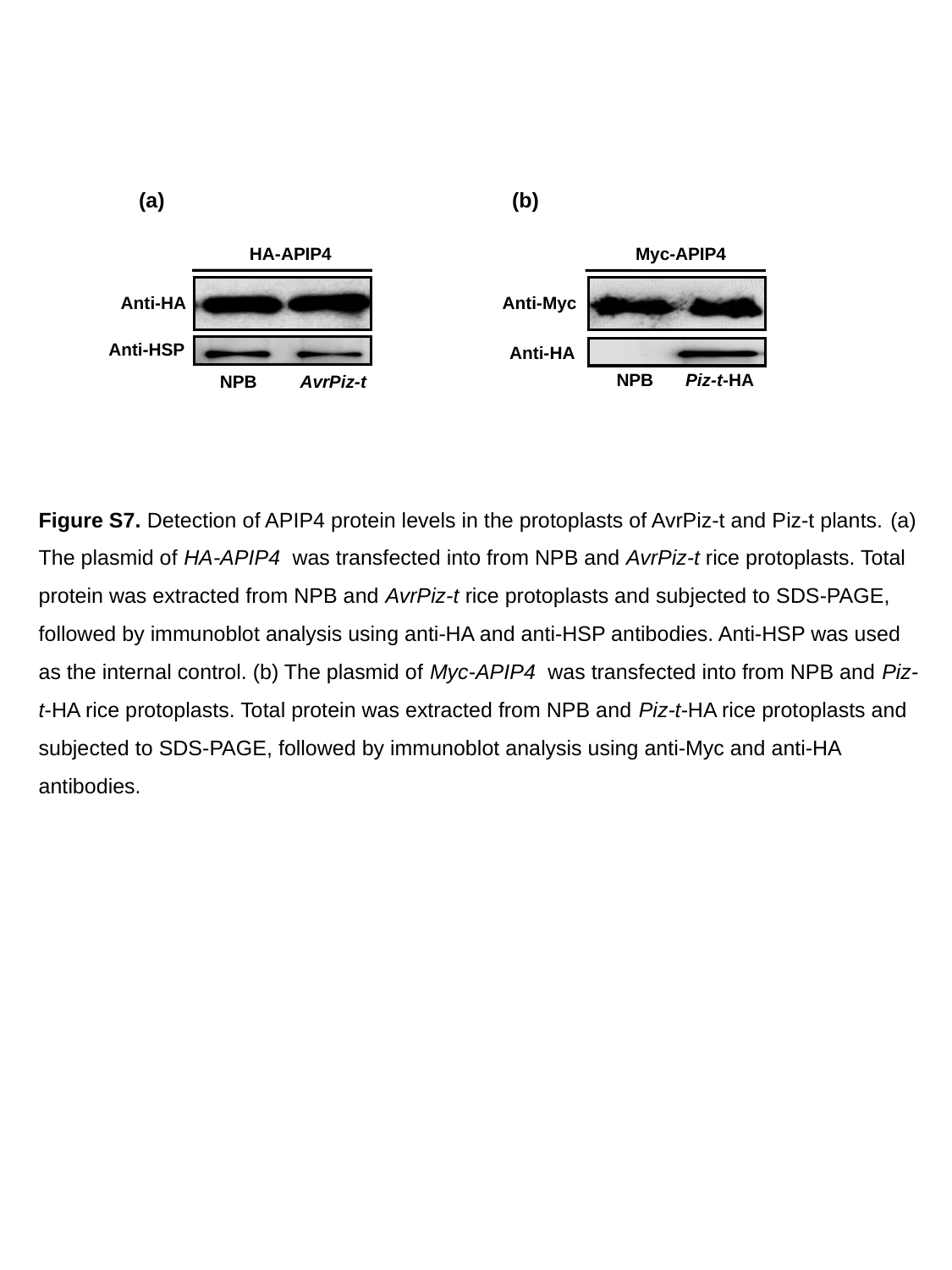

(a)
(b)
HA-APIP4
Myc-APIP4
Anti-HA
Anti-Myc
Anti-HSP
Anti-HA
NPB
Piz-t-HA
NPB
AvrPiz-t
Figure S7. Detection of APIP4 protein levels in the protoplasts of AvrPiz-t and Piz-t plants. (a) The plasmid of HA-APIP4 was transfected into from NPB and AvrPiz-t rice protoplasts. Total protein was extracted from NPB and AvrPiz-t rice protoplasts and subjected to SDS-PAGE, followed by immunoblot analysis using anti-HA and anti-HSP antibodies. Anti-HSP was used as the internal control. (b) The plasmid of Myc-APIP4 was transfected into from NPB and Piz-t-HA rice protoplasts. Total protein was extracted from NPB and Piz-t-HA rice protoplasts and subjected to SDS-PAGE, followed by immunoblot analysis using anti-Myc and anti-HA antibodies.

## Slide 8
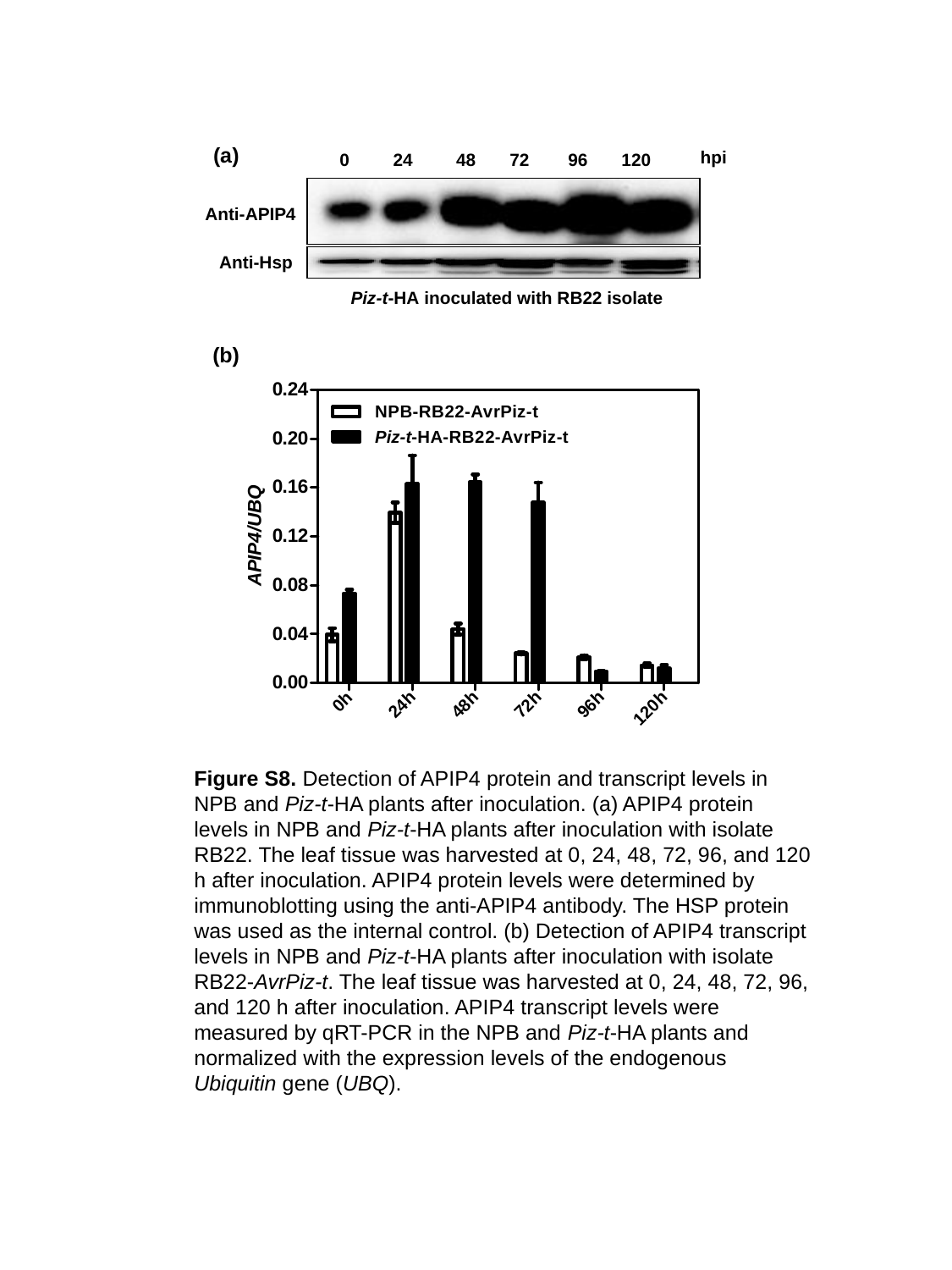

(a)
hpi
 0 24 48 72 96 120
Anti-APIP4
Anti-Hsp
Piz-t-HA inoculated with RB22 isolate
(b)
Figure S8. Detection of APIP4 protein and transcript levels in NPB and Piz-t-HA plants after inoculation. (a) APIP4 protein levels in NPB and Piz-t-HA plants after inoculation with isolate RB22. The leaf tissue was harvested at 0, 24, 48, 72, 96, and 120 h after inoculation. APIP4 protein levels were determined by immunoblotting using the anti-APIP4 antibody. The HSP protein was used as the internal control. (b) Detection of APIP4 transcript levels in NPB and Piz-t-HA plants after inoculation with isolate RB22-AvrPiz-t. The leaf tissue was harvested at 0, 24, 48, 72, 96, and 120 h after inoculation. APIP4 transcript levels were measured by qRT-PCR in the NPB and Piz-t-HA plants and normalized with the expression levels of the endogenous Ubiquitin gene (UBQ).

## Slide 9
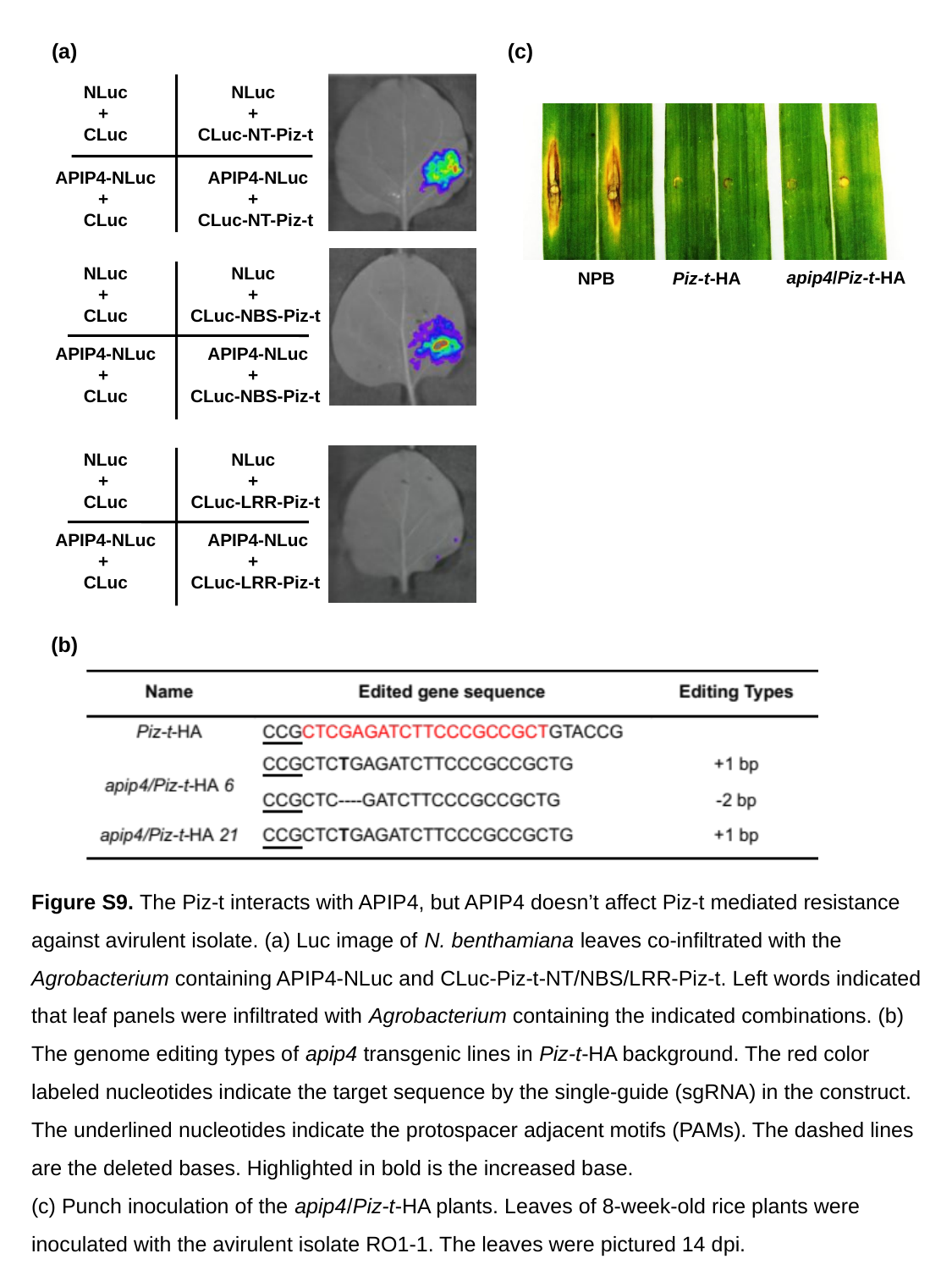

(a)
(c)
NLuc
+
CLuc
NLuc
+
CLuc-NT-Piz-t
APIP4-NLuc
+
CLuc
 APIP4-NLuc
+
CLuc-NT-Piz-t
NLuc
+
CLuc
NLuc
+
CLuc-NBS-Piz-t
apip4/Piz-t-HA
NPB
Piz-t-HA
APIP4-NLuc
+
CLuc
 APIP4-NLuc
+
CLuc-NBS-Piz-t
NLuc
+
CLuc
NLuc
+
CLuc-LRR-Piz-t
APIP4-NLuc
+
CLuc
 APIP4-NLuc
+
CLuc-LRR-Piz-t
(b)
Figure S9. The Piz-t interacts with APIP4, but APIP4 doesn’t affect Piz-t mediated resistance against avirulent isolate. (a) Luc image of N. benthamiana leaves co-infiltrated with the Agrobacterium containing APIP4-NLuc and CLuc-Piz-t-NT/NBS/LRR-Piz-t. Left words indicated that leaf panels were infiltrated with Agrobacterium containing the indicated combinations. (b) The genome editing types of apip4 transgenic lines in Piz-t-HA background. The red color labeled nucleotides indicate the target sequence by the single-guide (sgRNA) in the construct. The underlined nucleotides indicate the protospacer adjacent motifs (PAMs). The dashed lines are the deleted bases. Highlighted in bold is the increased base.
(c) Punch inoculation of the apip4/Piz-t-HA plants. Leaves of 8-week-old rice plants were inoculated with the avirulent isolate RO1-1. The leaves were pictured 14 dpi.

## Slide 10
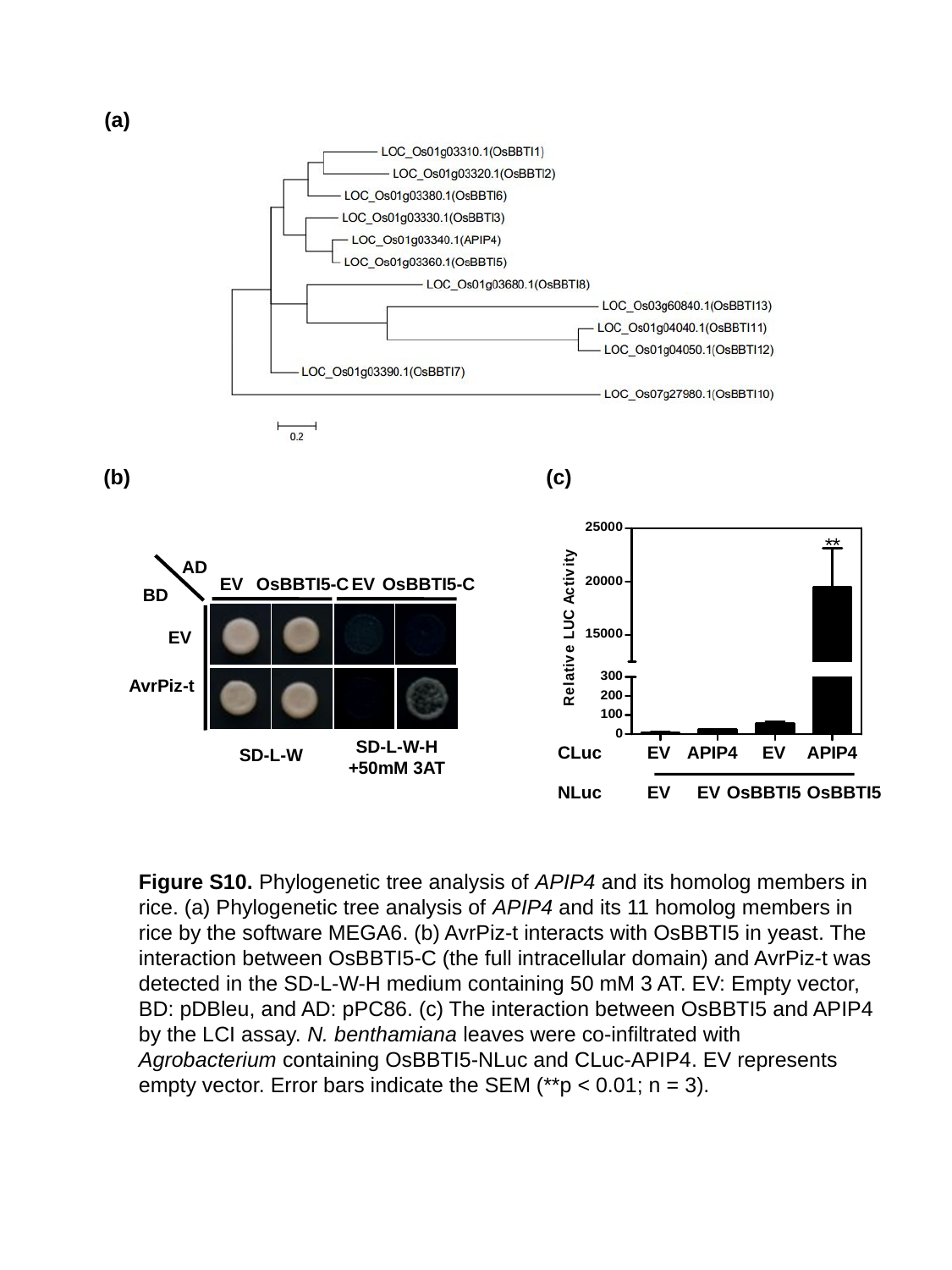

(a)
(b)
(c)
AD
EV
OsBBTI5-C
EV
OsBBTI5-C
BD
EV
AvrPiz-t
 SD-L-W-H
+50mM 3AT
CLuc
EV
APIP4
EV
APIP4
SD-L-W
NLuc
EV
EV
OsBBTI5
OsBBTI5
Figure S10. Phylogenetic tree analysis of APIP4 and its homolog members in rice. (a) Phylogenetic tree analysis of APIP4 and its 11 homolog members in rice by the software MEGA6. (b) AvrPiz-t interacts with OsBBTI5 in yeast. The interaction between OsBBTI5-C (the full intracellular domain) and AvrPiz-t was detected in the SD-L-W-H medium containing 50 mM 3 AT. EV: Empty vector, BD: pDBleu, and AD: pPC86. (c) The interaction between OsBBTI5 and APIP4 by the LCI assay. N. benthamiana leaves were co-infiltrated with Agrobacterium containing OsBBTI5-NLuc and CLuc-APIP4. EV represents empty vector. Error bars indicate the SEM (**p < 0.01; n = 3).
